# Small RNA regulation of ovule development in the cotton plant, *G. hirsutum *L

**DOI:** 10.1186/1471-2229-8-93

**Published:** 2008-09-16

**Authors:** Ibrokhim Y Abdurakhmonov, Eric J Devor, Zabardast T Buriev, Lingyan Huang, Abdusalom Makamov, Shukhrat E Shermatov, Tohir Bozorov, Fakhriddin N Kushanov, Gafurjon T Mavlonov, Abdusattor Abdukarimov

**Affiliations:** 1 Center of Genomic Technologies, Institute of Genetics and Plant Experimental Biology, Academy of Sciences of Uzbekistan. Yuqori Yuz, Qibray region Tashkent district, 111226 Uzbekistan; 2 Molecular Genetics, Integrated DNA Technologies, 1710 Commercial Park, Coralville, IA, 52241, USA

## Abstract

**Background:**

The involvement of small RNAs in cotton fiber development is under explored. The objective of this work was to directly clone, annotate, and analyze small RNAs of developing ovules to reveal the candidate small interfering RNA/microRNAs involved in cotton ovule and fiber development.

**Results:**

We cloned small RNA sequences from 0–10 days post anthesis (DPA) developing cotton ovules. A total of 6691 individual colonies were sequenced from 11 ovule small RNA libraries that yielded 2482 candidate small RNAs with a total of 583 unique sequence signatures. The majority (362, 62.1%) of these 583 sequences were 24 nt long with an additional 145 sequences (24.9%) in the 21 nt to 23 nt size range. Among all small RNA sequence signatures only three mirBase-confirmed plant microRNAs (miR172, miR390 and ath-miR853-like) were identified and only two miRNA-containing clones were recovered beyond 4 DPA. Further, among all of the small RNA sequences obtained from the small RNA pools in developing ovules, only 15 groups of sequences were observed in more than one DPA period. Of these, only five were present in more than two DPA periods. Two of these were miR-172 and miR-390 and a third was identified as 5.8S rRNA sequence. Thus, the vast majority of sequence signatures were expressed in only one DPA period and this included nearly all of the 24 nt sequences. Finally, we observed a distinct DPA-specific expression pattern among our clones based upon sequence abundance. Sequences occurring only once were far more likely to be seen in the 0 to 2 DPA periods while those occurring five or more times were the majority in later periods.

**Conclusion:**

This initial survey of small RNA sequences present in developing ovules in cotton indicates that fiber development is under complex small RNA regulation. Taken together, the results of this initial small RNA screen of developing cotton ovules is most consistent with a model, proposed by Baulcombe, that there are networks of small RNAs that are induced in a cascade fashion by the action of miRNAs and that the nature of these cascades can change from tissue to tissue and developmental stage to developmental stage.

## Background

Cotton (*Gossypium *spp.) ovule development is an interesting and unique developmental process because the differentiation and development of natural fiber, a seed epidermal trichome, occurs along with cottonseed embryogenic development [1]. Cotton is a very important natural textile fiber source, and cottonseed is a significant food source for humans and livestock [2]. Further, cotton fiber is not only an excellent single-celled model system to study cell elongation and cellulose biosynthesis in plants from a biological perspective, but also from a commercial perspective as the high cellulose content makes it an excellent biomass-material to produce ethanol-based biofuels [1,3]. Thus, molecular genetic studies of the stage-by-stage process of ovule development is important for understanding molecular mechanisms of fiber development and the goal of effective manipulation of fiber characteristics.

Cotton fiber is derived from a single cell. Fiber growth involves four overlapping developmental stages: fiber initiation, elongation (primary wall synthesis), wall thickening (secondary wall synthesis), and desiccation (maturation) [4]. Lint fiber initiation is conventionally timed at or just before anthesis [1]. Fiber initiation is a synchronous process that usually ends at 2 days post anthesis (DPA), but may extend up to 5 DPA [5]. This is then followed by an elongation stage at 5–20 DPAs, biosynthesis of secondary wall at 21–40 DPA, and maturation at approximately 40–60 DPA [1,6-8]. Fuzz fiber initiation and development occur after initiation of lint fiber development but this, too, is subject to variation from variety to variety [4,8].

Recent advances in cotton genomics [3,9] have led to the identification of the core genetic components contributing to the cotton fiber development and its molecular mechanisms [for reviews see [1,8]]. Although these studies have elucidated many aspects of fiber development and revealed important candidate genes expressed during developmental phases [5,7,8,10], many aspects of fiber cell differentiation remain unknown [11]. In particular, the potential role of small RNAs, including microRNAs (miRNAs) and endogenous silencing RNAs (esiRNAs), during ovule development remains to be determined [8]. Characterization of these small RNAs during the different stages of fiber development will contribute to identification of key molecular interactions that will, in turn, lead to better understand of the molecular mechanisms regulating cotton fiber development.

The universe of small RNAs, including both the 21 – 23 nt long miRNAs and the 24 nt long esiRNAs, has been expanding at an ever-increasing rate since their initial discovery in the early 1990s [12,13]. Small RNAs regulate their targets via transcriptional or posttranscriptional suppression either by DNA or histone modifications (esiRNAs) or direct cleavage of mRNAs and translational repression (miRNAs and siRNAs) [14]. The ~21 nt size class also includes trans-acting siRNAs (ta-siRNAs) that, unlike other siRNAs, potentially silence messages that are different from the RNAs from which they have been processed, but share some level of sequence similarity. Ta-siRNA biogenesis is mediated by specific miRNAs that process 21 snt size ta-siRNAs from TAS genes through direct cleavage [15].

The impact of small RNAs on a wide array of cellular processes in both plants and animals has grown to the point where it is becoming more and more difficult to find cellular processes that are not impacted by them to some degree. In plants, small RNAs have been implicated in processes as diverse as flowering [16,17] and overall cellular defense [18,19]. However, it is in development, such as fiber development in cotton, where small RNAs appear to have a major role [20,21]. For this reason, we have used a size-directed small RNA cloning strategy to isolate, clone, and sequence small RNAs expressed in eleven DPA periods of fiber development (0 – 10 DPA). We sequenced more than 6,500 clones and have identified nearly 2,500 candidate small RNAs. Among these candidates, we found 583 unique sequence signatures. Surprisingly, few of these candidates were identified as miRNAs in miRBase [22,23]. The majority of the sequences we found are, rather, the 24 nt long signatures corresponding to esiRNAs like those found in *Arabidopsis thaliana *and other plants. In addition, only 6.5% of identified small RNA sequences (or 8% in more saturated portion) were observed in more than one DPA of ovule development. While so-called "deep sequencing" using next generation platforms [24] will undoubtedly increase multi-DPA representation of small RNAs, our initial observations suggest that the initiation and elongation stages of cotton fiber-development are at least partially regulated by specific sets of small RNAs. Finally, target predictions based on ovule-derived small RNA sequences indicate involvement in numerous important biological processes including processes involving previously reported fiber-associated proteins.

## Results

### The small RNA profile of 0–10 DPA developing ovules

**Figure 1:**
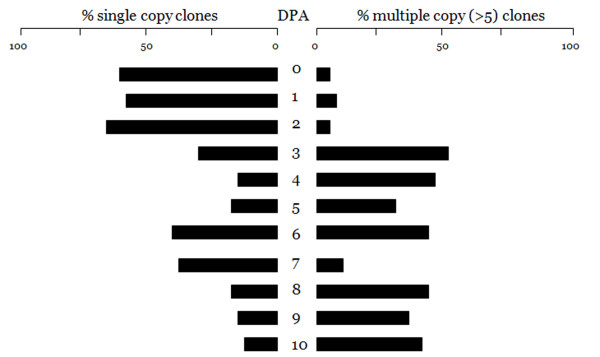
Proportionate distribution of small RNA sequences from single clones (left) and from five or more clones (right) by DPA period of cotton ovule development.

We sequenced a total of 6691 individual clones from the eleven *G. hirsutum *developing ovule libraries. From these clones we identified 2482 small RNAs having insert lengths between 12 nt and 39 nt. Following pooling of identical insert sequences, these 2482 clones yielded 583 unique sequence signatures. The distribution of small RNA sequence signatures is shown in Table 1. As can be seen, the majority of the unique sequence signatures, 507 of 583 (87%), lie in the 21 nt to 24 nt size range commonly associated with miRNAs and esiRNAs. Among these 21 nt to 24 nt small RNA sequences the majority, 362 of 507 (71%), correspond to the 24 nt length of putative esiRNAs. Further, a total of 259 (44%) unique small RNA sequences from 0 to 10 DPA were represented only once (Table 1; Additional file 1). We observed more sequence diversity, as indicated both by the total number of unique sequence signatures and by sequences only represented once, among small RNAs at the initial DPA periods of ovule development (0–2 DPA) than at the later DPAs of ovule development. At 0–2 DPA more than half of the unique sequence signatures were represented in only a single clone whereas at later DPA periods the percentage dropped to around 20% (Figure 1). The exception to this trend was seen in 6 and 7 DPA periods where just over 40% of the clones were singletons. The most disparate results relative to the other DPAs of ovule development were obtained at 9 DPA. Even though we sequenced more clones in this period compared to other DPA periods, we only recovered five unique signatures (Table 1, Additional file 1 and 2).

**Table 1:** Summary of small RNA pool in 0 to 10 days post anthesis (DPA) developing ovules of cotton, *G. hirsutum *L.

**Small RNA library**	No. of unique small RNA sequences*										**NUS (#)**	**GIS (#)**	**OTS (%)**	**NCS (#)**
					
	≥ 26 nt	25 nt	24 nt	23 nt	22 nt	21 nt	20 nt	19 nt	18 nt	17–12 nt				
0 dpa	2	5	72	16	7	3	1	1	-	4	111	223	58	576
1 dpa	2	6	58	12	5	1	1	-	-	3	88	203	53	480
2 dpa	3	4	79	20	12	9	3	-	2	1	133	276	61	747
3 dpa	-	2	20	2	2	3	-	3	-	2	34	325	29	530
4 dpa	-	4	25	4	-	1	1	-	-	-	35	241	20	506
5 dpa	1	-	28	13	7	2	1	-	-	1	53	288	23	708
6 dpa	-	2	13	5	3	1	1	1	-	-	26	233	42	354
7 dpa	1	3	14	2	1	2	2	-	1	1	27	216	41	663
8 dpa	1	3	21	4	1	-	-	-	-	2	32	208	22	779
9 dpa	1	-	1	1	1	-	-	-	-	1	5	48	20	927
10 dpa	-	1	31	4	1	-	-	1	-	1	39	221	18	421

Total	11	30	362	83	40	22	10	6	3	16	583	2482	44	6691

* #10 nt inserts were not included.

**NUS **– Number of unique small RNAs per library.

**GIS **– Number of all good insert sequences per each library (≥12 nt).

**OTS **– Number of one-time sequenced small RNAs (%) per each library.

**NCS **– Number of clones sequenced per each library.

Among the unique sequence signatures many were found in five or more different clones. These sequence signatures appear to represent abundantly processed and more or less saturated portion of small RNAs in each ovule library (see Additional file 3). These abundantly processed small RNAs are 21–25 nt long and the prevalent ones in each DPA period varied from 12 (1 DPA) to 144 (7 DPA) copies. The proportion of sequence signatureshaving ≥ 5 copies at each DPA period nearly mirrors the proportion of single copy sequences at that DPA (Figure 1). Moreover, there appears to be a distinct change in the pattern of small RNAs after 2 DPA. In 0–2 DPA, the majority of small RNAs are single copy and the proportion of sequences present in five or more clones is low. Beginning at 3DPA this pattern changes. Not only are there substantially fewer total sequence signatures (Table 1; Figure 1), the majority of sequences are seen in multiple clones as opposed to single clones. Only at 7 DPA is the early pattern seen to be present but it is represented by only 27 unique sequences. The combination of a great reduction in unique sequence signatures and in the ratio of single copy to multiple copy sequences following 2 DPA (Figure 1) suggests that there may be a change in the small RNA regulatory environment in cotton ovules once the early patterns are established.

Finally, in our data, only 6.5% (37 out of 583) of all cloned small RNAs were found to be expressed in two or more DPA periods (Table 2). Among these, ten sequences were found in two DPAs, one sequence in three DPAs, and four sequences were expressed in four DPA periods. Only 8% (11 out of 133) of abundant-copy small RNAs carried over in two or more DPAs. This further demonstrates a low level of small RNA carry-over from day-to-day ovule development because the abundant copy small RNAs represent more saturated pools of small RNAs in each day ovule library.

**Table 2:** Sequences of small RNAs expressed in two or more days post anthesis period (DPA) of cotton ovule development

Group#	Sequence ID#	Sequence (5'→3')	Length (nt)	#Clo-nes	dpa	MB/GB ID
1	Gh-sRNA-3dpa12	AAGAGGCUGUGUGGCUCACUGUGC	24	6	3	None
	Gh-sRNA-5dpq23	AAGAGGCUGUGUGGCUCACUGUGC	24	4	5	
2	Gh-sRNA-3dpa19	AGGGUGAGCGUUUGAUUGAGUUGA	24	1	3	None
	Gh-sRNA-4dpa6	AGGGUGAGCGUUUGAUUGAGUUGA	24	8	4	
3	Gh-sRNA-3dpa 24	UGCCCUUCAAUAUCACAAGUGC	22	3	3	None
	Gh-sRNA-6dpa21	UGCCCUUCAAUAUCACAAGUGC	22	3	6	
4	Gh-sRNA-4dpa25	**UUG**AGAAUCCUGAUGAUGUUGCAG	24	1	4	miR-172
	Gh-sRNA-0dpa103	AGAAUC**U**UGAUGAUGCUGCAG	21	4	0	
	Gh-sRNA-3dpa26	AGAAUCCUGAUGAUGCUGCAG	21	9	3	
	Gh-sRNA-2dpa119	AGAAUCCUGAUGAUGCUGCAG	21	13	2	
5	Gh-sRNA-3dpa29	AAAUCGUGCCCUAACGUAUUGAGU	24	1	3	None
	Gh-sRNA-4dpa12	AAAUCGUGCCCUAACGUAUUGAGU	24	15	4	
6	Gh-sRNA-3dpa3	AGGUCAUGAGAGGCCCACAUGAGC	24	11	3	None
	Gh-sRNA-8dpa6	AGGUCAUGAGAGGCCCACAUGAGC	24	11	8	
7	Gh-sRNA-3dpa4	UUUUUCACUGUCCAAGGUAAGCCU	24	5	3	None
	Gh-sRNA-6dpa9	UUUUUCACUGUCCAAGGUAAGCCU	24	17	6	
	Gh-sRNA-7dpa15	UUUUUCACUGUCCAAGGUAAGCCU	24	3	7	
	Gh-sRNA-10dpa2	UUUUUCACUGUCCAAGGUAAGCCU	24	20	10	
8	Gh-sRNA-10dpa12	AGUGUCACGGAACAAAUGUCUUGA	24	5	10	None
	Gh-sRNA-4dpa2	AGUGUCACGGAACAAAUGUCUUGA**U**	25	12	4	
9	Gh-sRNA-4dpa24	AUCAAAGCCCAUGACAAAUGCACA	24	2	4	None
	Gh-sRNA-7dpa18	AUCAAAGCCCAUGACAAAUGCACA	24	1	7	
10	Gh-sRNA-4dpa30	**G**CACGUCUGCCUGGGUGUCACGC	23	16	4	5.8S rRNA
	Gh-sRNA-2dpa105	**U**C**G**CGUCUGCCUGGGUGUCACGC	23	1	2	
	Gh-sRNA-8dpa27	**G**CACGUCUGCCUGGGUGUCACGC	23	9	8	
	Gh-sRNA-0dpa100	CACGUCUGCCUGGGUGUCACGC	22	1	0	
11	Gh-sRNA-4dpa9	AAAUGAUAGGCUUGCCCGGGUGGU	24	11	4	None
	Gh-sRNA-7dpa17	AAAUGAUAGGCUUGCCCGGGUGGU	24	1	7	
12	Gh-sRNA-2dpa38	AACCUGCAUCUCCACCUUAUUAUU	24	8	2	None
	Gh-sRNA-5dpa02	AACCUGCAUCUCCACCUUAUUAUU	24	2	5	
	Gh-sRNA-1dpa9	AACCUGCAUCUCCACCUUAUUAUU	24	3	1	
13	Gh-sRNA-2dpa104	**GAGC**CAAAAUGAGAUAGAUAAGC	23	1	2	None
	Gh-sRNA-5dpa31	CAAAAUGAGAUAGAUAAGC**UGAA**	23	3	5	
14	Gh-sRNA-7dpa23	AAGCUCAGGAGGGAUAGCGCC	21	2	7	miR-390
	Gh-sRNA-1dpa84	AAGCUCAGGAGGGAUAGCGCC	21	4	1	
	Gh-sRNA-0dpa104	AAGCUC**G**GGAGGGAUAGCGCC	21	1	0	
	Gh-sRNA-2dpa126	AAGCUCAGGAGGGAUAGCGCC	21	1	2	
15	Gh-sRNA-2dpa30	GAUCGACCCGAUU**U**AAGCAACGAA	24	2	2	None
	Gh-sRNA-0dpa12	GAUCGACCCGAUU-AAGCAACGAA**C**	24	2	0	

MB/GB-mirBase/GenBank; different nucleotides in mature sequences are shown in bold-faced letters.

### BLAST similarity search of ovule derived small RNAs

Although most of the small RNA sequences in the ovule libraries did not match with any known genes or ESTs in BLAST analyses, several small RNAs were found to have a significant (e≤0.01) nucleotide identity homologous to *Gossypium *derived cDNA, including ESTs, sequenced from developing ovule library as well as ribosomal and chloroplast DNA (Additional file 1). In addition, in specific DPA periods, we found significant putative matches with known genes such as alcohol dehydrogenase A gene of *Gossypium*, FAD-dependent oxidoreductase, disulfide isomerase genes, transposons and retrotransposon elements, peroxisome proliferator activated receptor, amino acid permease, armadillo/beta-catenin repeat containing protein (s), protein kinases, F-box protein family, transcription factors, and anion exchange proteins (see Additional file 1). Blast analysis identified only three groups of small RNA signatures, overlapping at two or more DPAs. These three were all seen to be expressed in four different DPA period and two were identified as plant miRNAs; miR-172 and miR-390. The third sequence was identified as a fragment of 5.8S rRNA. None of the other putative esiRNA 24-mers expressed in multiple DPA periods could be identified (Table 2).

### MiRBase database search

Because BLAST searches identified two known plant miRNAs among the sequences expressed in two or more DPA periods, we screened all cotton ovule-derived small RNAs in miRBase in an effort to identify additional miRNA sequences. Surprisingly, only two families of miRBase-confirmed plant miRNAs were identified in the 583 sequences and these were the miR-172 and miR-390 miRNAs already identified in Table 2. No other definite microRNAs were found. One similar sequence was detected in a 24-mer represented in 21 clones at 3 DPA. This sequence was roughly similar to miR-853 from *Arabidopsis thaliana *[25] but not similar enough to make a call one way or the other.

The putative expression profiles of miR-172 and miR-390 shown in Table 2 are consistent with the pattern shown in Figure 1. Both miR172 and miR390 are predominantly expressed during the earliest DPAs of ovule development. Moreover, there are a few mature sequence differences among the clones that are consistently replicated in within-DPA copies (Table 3). All of the miR-172 clones from 0 DPA and all but the 0 DPA miR-390 clones display perfect matches with canonical mature sequences from miRBase. The U→C change in position 7 of miR-172 observed in a total of 22 signatures of 2 DPA and 3 DPA clones is a perfect match for miR-172i reported in the black cottonwood, *Populus trichocarpa*, [26]. The additional C→U change at position 16 in the single 4 DPA clone does not match any miR172 variants. Similarly, the A→G change seen at position 7 of the single 0 DPA miR-390 clone fails to match any known variant. Both of these single clone sequence differences could simply be sequencing errors whereas the miR-172i variant is too well supported to be dismissed as a sequencing error. Also shown in Table 3 are mir-172 and miR-390 sequences reported for cotton by Zhang et al. [11] and a miR-390 cotton sequence reported by Qui et al. [27]. The Qui et al. sequence is canonical but the Zhang et al. sequences are very poor matches to any plant microRNA.

**Table 3:** Comparison of mature microRNA sequences of developing ovules with published cotton microRNAs

microRNA	Copies	Sequence 5'-3'	Reference
0 DPA miR-172	4	AGAAUC**U**UGAUGAUGCUGCAG	This study
2 DPA miR-172	13	AGAAUCCUGAUGAUGCUGCAG	This study
3 DPA miR-172	9	AGAAUCCUGAUGAUGCUGCAG	This study
4 DPA miR-172	1	**UUG**AGAAUCCUGAUGAUG**U**UGCAG	This study
172a		**U**GAAUCUUGAUGAUG**CCAA**A**U**	Zhang et al. [11]
172b		**AUCGCACC**AU**U**A**A**G**AUUACU**	Zhang et al. [11]
172c		**CCAGCACC**AU**U**A**A**G**AAUC**AG	Zhang et al. [11]
0 DPA miR-390	1	AAGCUC**G**GGAGGGAUAGCGCC	This study
1 DPA miR-390	4	AAGCUCAGGAGGGAUAGCGCC	This study
2 DPA miR-390	1	AAGCUCAGGAGGGAUAGCGCC	This study
7 DPA miR-390	2	AAGCUCAGGAGGGAUAGCGCC	This study
390		AAGCUCAGGAGGGAUAGCGCC	Qui et al. [27]
390		A**GU**CUCAGGAGGGAUAGC**UU**C	Zhang et al. [11]

Different nucleotides in mature sequences are shown in bold-faced letters.

### Putative small RNA targets

Analysis of the 583 small RNA sequence signatures from cotton ovule using Target Finder [28,29] identified a total of 871 possible protein targets of these small RNAs (Additional files 1 and 3, 4, 5, 6, 7). Consistent with the distribution of the sequence signatures themselves, a number of these putative protein targets were specific to one day of ovule development (Table 4). Also consistent with the pattern of small RNA expression, the relative occurrence of DPA-specific potential protein targets appears to change between 2 DPA and 3 DPA as their representation drops from about one-third to one-quarter of potential targets identified and the total number of targets drops off.

**Table 4:** Distribution of potential protein targets by DPA period of cotton ovule development

**Developmental period**	**Total targets#**	**DPA-specific targets# (%)**	*Multi-DPA targets# (%)
0 DPA	326	108 (33.1)	218 (66.9)
1 DPA	256	81 (31.6)	175 (68.4)
2 DPA	326	112 (34.4)	214 (65.6)
3 DPA	92	13 (14.1)	79 (85.9)
4 DPA	116	24 (20.7)	92 (79.3)
5 DPA	182	46 (25.3)	136 (74.7)
6 DPA	85	15 (17.7)	70 (82.3)
7 DPA	95	17 (17.9)	78 (82.1)
8 DPA	118	28 (23.7)	90 (76.3)
9 DPA	53	12 (22.6)	41 (77.4)
10 DPA	164	39 (23.8)	125 (76.2)
Total	1813	495 (27.3)	1318 (72.7)

*#potential protein targets identifies in more than one DPA period

While such analyses are purely speculative at this early stage, an assessment of putative protein targets of our small RNA sequences, based on their function Gene Ontology (GO) molecular function and biological process from TAIR database, indicates that they are involved in numerous biological processes. These processes, which include biosynthesis/metabolism, transport, cell growth and organogenesis, gene regulation, photomorphogenesis, response to phytohormones, response to biotic/abiotic stresses, disease resistance, and DNA biogenesis (see Additional files 1 and 3, 4, 5, 6, 7), are clearly congruent with what would be expected in a developmental phenomenon like that examined here. The putative protein target information for mirBase confirmed ovule miRNA signatures are also listed in Additional file 8, demonstrating that these miRNAs putatively regulate important proteins involved in transcription (e.g. AP2, C3HC4, MYB68, and DPB1) and translation (eIF-4A), metabolism and biosynthesis (e.g. SDR, DXP, FPS1), transport of cations and protons (CHX), disease resistance (LRR), cytoskeleton components (myosin), ribosomal proteins (RPS15aE), response to light stimulus (GRL1.1), and DNA biogenesis (DNA polymerase III; see Additional file 7 for abbreviated protein names).

## Discussion

We have cloned and sequenced small RNAs derived from eleven DPA periods (0–10 DPA) of cotton fiber development. Our results support the potential importance of small RNAs in developing cotton ovules. Overall, we find that small RNA sequences are more diverse and abundant in early development periods (0–2 DPA) than in subsequent periods (3–10 DPA). This suggests that the genetic processes regulated by small RNAs in the initiation phases of ovule and fiber development are at least qualitatively different than those at later periods. Whether this says that those early genetic, physiological, and biochemical mechanisms are more complex in the initiation phase than they are in other stages like the elongation phase is unknown at this point. However, it remains that the small RNAs in our study were very diverse: 44% of them were represented by small RNAs sequenced only once and that the majority of these were found in the earliest DPAs of development. This is similar to the case of Arabidopsis, in which 65% of all unique small RNAs were sequenced only once [25]. Although the material and overall approach were different from what we did here, unique small RNAs represented ~38% of total reads in a genome-wide survey in Arabidopsis [25] while the unique small RNA sequences in cotton ovules represented ~23% in our study. In addition, the fact that only a small number of unique candidate small RNA sequences spanned two or more DPA periods suggests that small RNA regulation in each DPA period in cotton is different. Perhaps, small RNA regulation in each DPA is highly specific and that shifting controlling biological processes occurs quite rapidly between days in ovule development.

A surprising finding in our study is that, out of 583 candidate small RNA sequences from 0–10 DPA developing ovule tissues, only two plant miRNA families (miR172 and miR390) were confirmed in miRBase. There is evidence that miRNAs constitute a much smaller proportion of the small RNAs in plants than in animals [30]. Our data are clearly consistent with this view. However, in our data, there are many other 21- to 23-mer small RNA sequences that do not match any of the currently miRBase-annotated plant or other organism miRNAs. These are potential candidates for new cotton-specific miRNAs, which need to be further explored.

Replicated sequence differences observed in the mature sequence of both miR172 and miR-390 suggest the existence of multiple miR172 and miR-390 family members in cotton, and potentially, there are different miR172 and miR-390 members functioning at different DPA periods. The miRBase confirmed miRNAs putatively target proteins that may play an important role both in ovule embryonic and in fiber development. Proteins putatively targeted by miR172 and miR390 include MYB and zinc finger transcription factors, glycosyl transferease family proteins, action/hydrogen exchanger, translation initiation factors (eIF-4A), and myosin heavy chain proteins. These proteins have been reported to be associated with fiber development, including being important components in gene regulation, in cytoskeleton and cellulose synthesis, and in proton and cation transporting [5,7,8]. In addition, although experimental validations are needed, other putative target proteins of miR172 may also be involved in the fiber development process. For example, short chain dehydrogenase/reductase (SDR) which is targeted by miR172 at 0 DPA. SDR has cellulose and pectin containing cell wall oxireductase activity and is involved in ABA biosynthesis, in which ABA is considered to be an important phytochormone in fiber development [8]. Additional proteins targeted by miR172 that are likely candidate proteins affecting fiber development include phosphoenolpyruvate carboxylase [31], the glutamate receptor involved in dendritic cell growth [32], and YT521-B-like family proteins changing alternative splice site usage in concentration dependent manner [33].

In Arabidopsis, miR390 was reported to target *TAS3 *trans-acting siRNA (ta-siRNA) biogenesis through coupling with AGRONAUTE7 (AGO7) and regulating AUXIN RESPONSE FACTOR3 (ARF3). Identification of at least two miR390s, of which, one is expressed at 7 DPA, is good evidence supporting specific involvement of miR390 in fiber development as auxin response is one of the most important factors in fiber initiation and elongation in cotton [8]. In addition, since the majority of ta-siRNAs are 24 nt long and are hypothesized to be generated in miRNA-induced cascades [34,35], it is possible that miRNAs that are expressed at different DPAs of ovule development are the headwaters of within-DPA ta-siRNA cascades that may be indicated here by the abundance of unique 24-mer RNAs in the various DPAs.

Although structurally characterized [25,36], the function of miR-853 is not clear in the literature. The ovule-derived candidate miR853-like small RNA targets several unknown proteins in Arabidopsis and cotton gene index databases. Arabidopsis ath-miR853 matches several putative targets in cotton gene index database. Of those, both extensin-like proteins and RAS-related proteins are known to be involved in fiber development [8,37,38]. In addition, a palmitoyl – acyl carrier protein thioesterase, catalyzing the palmitic acid of the fatty acid family in plants [39], could be important. The role of fatty acids (FA) and very long chain fatty acids (VLCFA) in fiber development has been reported [40-43]. This suggests that miR853-like small RNAs play a role in fiber development of cotton, possibly as miRNAs, and requires further study.

Other small RNAs in both the total or in the abundant copy portion targeted many *a priori *fiber development-associated proteins that have been reported in previous studies. Ovule-derived candidate small RNAs, putatively targeted many transcription and translation factors, biosynthesis/metabolism (catabolism), hormone mediated signal transduction pathways, and hormone responsive proteins and factors of all key plant phytochormones such as IAA, ABA, GA, BR, ethylene and cytokinin, which are known to be the key factors associated with fiber development [1,8]. Other important fiber-associated factors reported in the literature are involved in the transportation of proteins, carbohydrates, lipids, ions, and electrons [7,44-47], in lipid and fatty acids biosynthesis/metabolism [40-43], in cytoskleton formation [1,5,7,8,10,48], in peroxidase activity [49], in carbohydrate biosynthesis/metabolism [1,45,47,50-53], and in DNA biogenesis (e.g. endoreduplication) [1,8,54]. Those factors were also found to be targeted by candidate small RNAs in cotton ovules in our study. It is noteworthy to mention that ovule-derived candidate small RNAs also target some of the recently highlighted proteins such as prohibitin and steroid sulfotransferase, MATE efflux protein, and transducin family proteins that are differentially expressed in fiber initials [5], and actin depolymerizing factors (ADF). Some of which, like GhADF2, are predominantly expressed in fiber tissue [55]. Others, like the dynamin family proteins, are differentially expressed in fibers cells at 15 DPA period of the superior quality chromosome substitution line CSB22sh [7]. Recently, MADS-box genes [56] and genes of vesicle coating and trafficking [57] were found to be associated with fiber development where these related proteins are targeted by ovule-derived small RNAs annotated in this study.

Although *in silico *target predictions have been shown a good tool to putatively annotate the small RNA functions [29], the experimental validation of the exact biological functions of small RNAs in plant cells is necessary. Transient expression systems using *in vitro *ovule culture [1] with these candidate small RNAs may provide a valuable tool for rapid validation of small RNAs and miRNAs. Cloning and characterization of small RNAs selectively from the fiber cells of developing ovules using a newly developed methodology [5] should efficiently facilitate the identification of fiber-specific small RNAs/miRNAs in cotton and differentiation of fiber-specific [5] versus ovule specific [58] small RNA signatures. In addition, characterization of small RNAs/miRNAs from fiber mutants such as naked seed (*n*_1_), Ligon lintless (*Li*_1_*, L*_2_), pilose mutant (*H*_2_), immature fiber mutant (*im*), and other fiber mutants with distinctive fiber development may help to identify key small RNAs/miRNAs affected by these variations and further elucidate the mechanisms of the fiber development process. Annotation of small RNA pools from the remaining ovule and fiber development stages (10–50 DPA) will also be important for the understanding of developmental processes such as the secondary wall deposition and maturation stages of fiber development [48]. Consequently, with the availability of complete cotton genome sequences [9] in a near future, mapping of these siRNAs throughout the cotton genome will facilitate studies of structural and functional processes, biogenesis, and evolution of these ovule-derived small RNAs/miRNAs in cotton. These all require further attention and efforts on comprehensive studying of small RNA world of complex fiber development process in cotton.

## Conclusion

We have carried out an initial survey of small RNA species expressed during cotton ovule development from 0 DPA to 10 DPA. Our results provide initial evidence of extensive small RNA-mediated regulation of complex ovule and fiber development processes in cotton. The majority of small RNA sequence signatures observed corresponds to the 24 nt size characteristic of endogenous silencing RNAs (esiRNAs), and there is very little carry over between DPA periods. Where there is DPA-to-DPA carry over, those sequences that have been identified are plant miRNAs. The observation that there are considerably more different sequence signatures present in the earliest DPAs of ovule development (0 DPA to 2 DPA) than in later periods may indicate that there is a shift in the regulatory landscape after 2DPA with fewer small RNAs present but in higher numbers later on. The overall patterns of small RNA expression observed raise the possibility that this regulation is consistent with miRNA-initiated small RNA regulatory cascades potentially targeting a large number of previously known fiber-associated proteins as well as previously unknown targets. Confirmation of our results, in particular the three miRBase-confirmed plant miRNAs and a the large number of 24 nt esiRNAs putatively involved in fiber initiation and elongation stages of ovule development, by ongoing deep sequencing efforts will greatly facilitate understanding of the developmental mechanisms involved.

## Methods

### Plant material

Zero to ten DPA ovule tissues were collected from *G. hirsutum *var. C9080 cultivar, which have superior fiber quality and is one of the commercialized varieties in Uzbekistan. Plants were grown in the standard cultivation conditions at the field station of the Institute of Genetics and Plant Experimental Biology. Flowers were tagged with papers before the day of anthesis and ovules were collected each day following the day of anthesis. Collected ovule tissues were immediately frozen in liquid nitrogen on site and were stored at -80° C until RNA isolation.

### Small RNA isolation and cloning

Ovule tissues were placed into RNA Later Ice™ solution (Ambion, USA) a day before RNA isolation and stored at -20°C overnight. A total RNA pool was isolated from RNA Later Ice™-treated cotton ovule tissues using the mirVana RNA isolation kit (Ambion, USA) per the manufacturer's guidelines. RNA quality and relative yields were checked on 15% denaturing (7 M Urea) polyacrylamide gels (dPAGE). The small RNA fraction (15 nt -25 nt) was isolated from dPAGE gel slices. The desired RNA size was identified in the gels using an internal 21 nt long RNA marker (miSPIKE™, Integrated DNA Technologies, USA). Small RNAs were purified from the gel slices using a standard crush and soak method in a cold ethanol bath, desalted using Biogel P-30 spin columns (BioRad, USA), and vacuum dried. The purified small RNAs were cloned using the miRCat™ small RNA cloning kit (Integrated DNA Technologies, USA). Briefly, purified small RNAs are 3' ligated to an adenylated cloning linker containing a 3' block (5'-rAppCTGTAGGCACCATCAATddC-3') using T4 RNA Ligase in the absence of ATP. Ligations are carried out at 22°C for two hours. Ligated RNAs are purified by a second round of dPAGE and then 5' ligated with a DNA/RNA chimeric linker (5'-TGGAATucucgggcaccaaggu-3') using T4 RNA Ligase in the presence of 10 mg/ml ATP. Doubly linkered RNAs were reverse transcribed with a 3' linker-specific reverse primer (5'-GATTGATGGTGCCTACAG-3', Tm = 50.2°C).

PCR amplification of the reverse transcripts was carried out using the RT primer as the reverse primer and a linker-specific forward primer (5'-TGGAATTCTCGGGCACC-3', Tm = 55.0°C). PCR conditions were 95.0°C for five minutes followed by 25 cycles of 95.0°C for 30 seconds, 52.0°C for 30 seconds, and 72°C for 30 seconds and finishing with a final extension step of 72.0°C for seven minutes. PCR amplicons in the expected range of 60–65 bp were obtained, further gel purified, and then cloned into TOPO-TA cloning vectors and transformed into TOP10 one-shot *Escherichia coli *cells according to manufacturer's instructions (Invitrogen, USA). *E. coli *transformants were spread and grown on LB plates containing 50 mg/ml kanamycin over-night. A detailed protocol for miRCat™ can be found on-line at Integrated DNA Technology website [59].

### Colony PCR

The *E. coli *transformants were screened for inserted PCR-products by colony-PCR using universal M13 forward and reverse primer pairs. Amplification reactions were performed in 50 μl volumes containing 4.5 μl 10 × PCR buffer with MgCl2, 1μl BSA, 0.5 μl 25 mM of a dATP, dGTP, dTTP, and dCTP mix, 2.5 μl 50 ng/ml of each reverse and forward primer, and 0.5 U Taq DNA polymerase (Sigma, USA). Afterward, bacterial cells from the 4–5 mm sized bacterial colonies were dipped into PCR cocktail using tooth picks. Amplifications were carried out with first denaturation at 96°C for 3 min followed by 45 cycles of 94°C for 1 min, 55°C for 1 min (annealing), and 72°C for 2 min (extension). A final 5-min extension at 72°C was then performed. PCR products were verified by 2%-agarose (Sigma; USA) gel-electrophoresis in 0.5 × TBE buffer. Gels were then visualized with ethiduim bromide.

### Sequencing

PCR products representing individual colonies were precipitated using a PEG (26% PEG 8000, 6.5 mM magnesium chloride, 0.6 M sodium acetate, pH 6–7) solution. The purified amplicons were re-suspended in 10 mM Tris-EDTA (TE) buffer to be used as the templates for sequencing. More then 350 (an average of ~600 colonies per each ovule library) positive clones were sequenced from each of the eleven ovule libraries to maximizing the coverage of the small RNA content of each DPA period. Cycle sequencing was performed on the GeneAmp PCR System 9700 (Applied Biosystems, USA) using the ABI PRISM™ Big Dye terminator (Applied Biosystems, CA, USA). Briefly, PCR amplification was performed in 10 μl reaction mix containing 1 μl 50 ng/ml of sequencing primer M13 and 2 μl recombinant plasmid with 4 μl of premixture (containing buffer, dNTPs, dye-labeled ddNTPs and Taq-FS/pyrophosphatase). After the initial denaturation at 96°C for 1 min, the reaction was incubated for 35 cycles of 95°C for 30 sec, 55°C for 15 sec and 60°C for 4 min. Excess dye-labeled terminators were removed from the extension products by standard ethanol precipitation methods (Applied Biosystems, CA, USA). Once separated, the extension products were dried down. Samples were re-suspended in 10 μl of Hi-Di™ formamide solution. Aliquots of the extension products were loaded onto the ABI Genetic Analyzer 3100xl (Applied Biosystems, USA).

### Data analyses

Small RNA sequences were analyzed in Sequencher 4.5 (Gene Codes, USA) where vector and linker sequences trimmed and appropriate 3' and 5' ends of inserted small RNAs were defined based on linkers. To annotate these candidate siRNAs, sequences were blasted against GenBank [60], TAIR Higher plant EST database [61], Cotton Pilot Project [62], and miRBase [63]. The putative target analysis performed with Target Finder [28,29] using the *A. thaliana *TIGR mRNA (TIGR Ath1_5) database. The TIGR rice (*Oryza staiva*) genome mRNA database was used for evolutionary conservation comparison. TIGR Cotton Gene Index 6 database is also used in some cases when TIGR Ath1_5 did not find any matched target proteins. To simplify the analyses, targeted protein members of the same protein family were, first, unified in each DPA and then, pooled to identify specific and overlapping targets at different DPA periods. GO molecular function and biological process of these putative targets of unified list was then analyzed using TAIR protein database information.

### Accession numbers

Sequence data from this article can be found in the GenBank data library under accession numbers [GenBank: EU540624 – EU541206].

## Authors' contributions

**IYA **designed overall experiments; performed cloning experiments, analyzed the entire data, interpreted the results, wrote the manuscript, and made final revision; **EJD **significantly contributed to this work by developing the cloning methodology, providing cloning kits for experiments, analyzing the data, interpreting the results and editing and revising the manuscript; **ZTB **performed total RNA isolation and sequencing experiments, helped with manuscript data preparation; **LH **was instrumental in small RNA cloning methodology development, and in editing and revising the manuscript; **AM **performed ovule tissue collection, extensively helped with siRNA cloning and sequencing; **SES **performed sequencing of colonies and helped with annotation of siRNAs and data preparation; **TB, FNK, and GTM **helped with annotation of GO molecular and biological function and data organization for analyses; **AA **designed the experiments, interpreted the results, edited and approved the manuscript for publication. All authors read and approved the final manuscript.

## Supplementary Material

Additional file 1 **Small RNA pools and their targets of developing ovules of cotton at zero to ten days post anthesis (DPA) periods (detail).** Click here for file

Additional file 2 **Bar graphs for small RNA species cloned from 0–10 DPA ovules. Different sized small RNAs are color-coded.** Click here for file

Additional file 3 **Detail putative targets of high-copy small RNAs (> 5 copies) in developing ovules of cotton.** Click here for file

Additional file 4 **Putative target proteins for small RNAs of 0 to 10 dpa developing ovule libraries.** **Note: Some of the same family protein members were unified; see the detail list of targets for each siRNA in Additional file 1, including GenBank ID and target score for each protein. Click here for file

Additional file 5 **Putative target proteins for abundant copy small RNAs of 0–10 dpa developing ovule libraries**. **Note: Some of the same family protein members were unified; see the detail list of targets for each siRNA in Additional file 3, including GenBank ID and target score for each protein. Click here for file

Additional file 6 **The schematic representation of fiber development stages and putatively targeted proteins by siRNAs of each 0–10 DPA ovules.** Click here for file

Additional file 7 **The list of abbreviated putative target proteins (partial) used in Additional file 6 and the text.** Click here for file

Additional file 8 **MirBase confirmed microRNAs functioning in different DPAs of cotton ovule development.** * Blasted against GenBank (NCBI); TAIR (AGI and Higher plant EST databases); and Cotton Pilot Project (CPP) EST database; **In parentheses, the target scores and conservation (y) between *A. thaliana *and *O. sativa *genomes were given. Click here for file
