# Supplementary figures and images for: Small RNA regulation of ovule development in the cotton plant, G. hirsutum L
Source: BMC Plant Biol. 2008 Sep 16;8:93. doi: 10.1186/1471-2229-8-93 (PMC2564936; doi:10.1186/1471-2229-8-93)

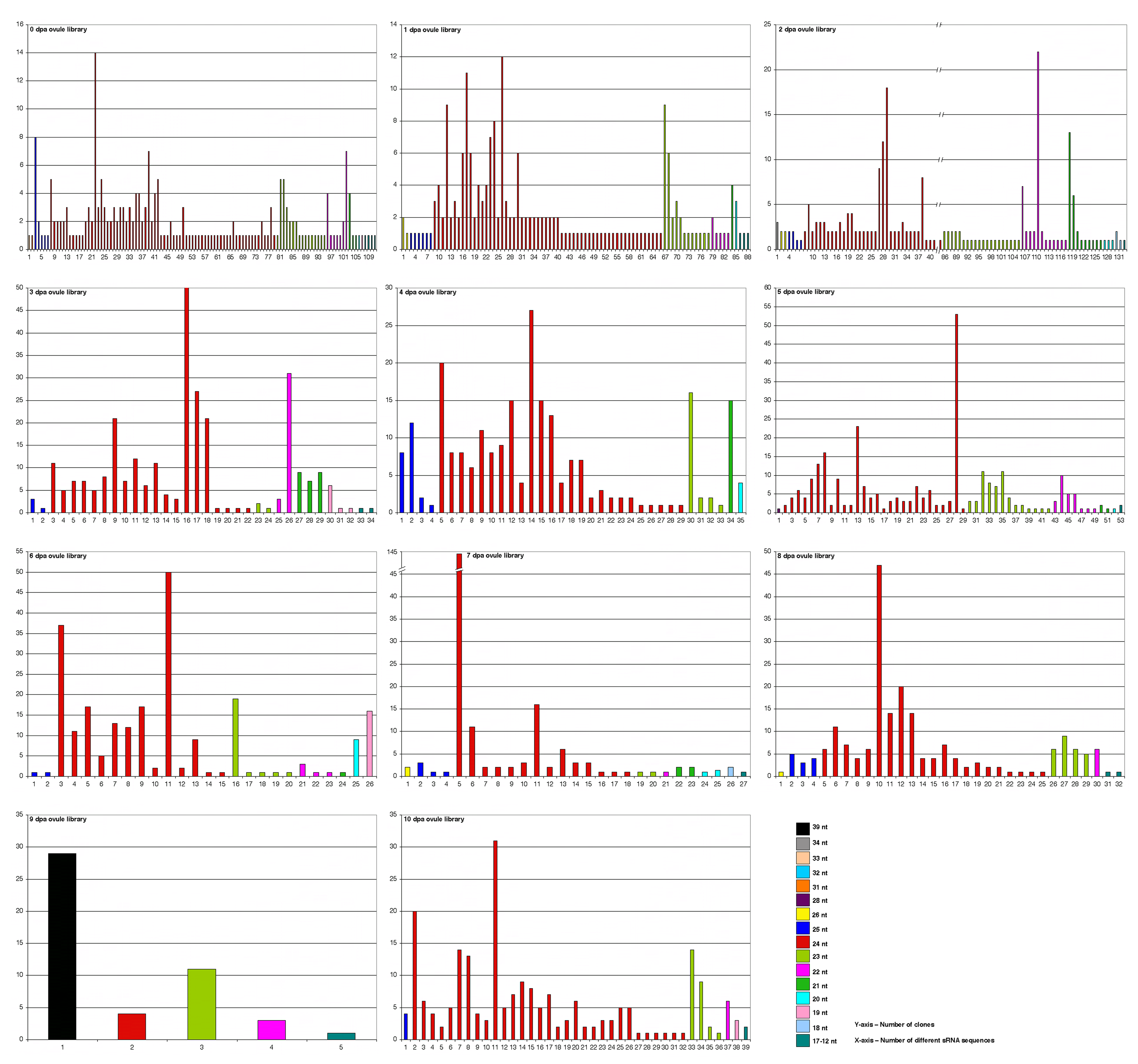

Supplement: Additional file 2 — Bar graphs for small RNA species cloned from 0–10 DPA ovules. Different sized small RNAs are color-coded. [file 1471-2229-8-93-S2.tiff]
