# Supplementary material for: Small RNA regulation of ovule development in the cotton plant, G. hirsutum L
Source: BMC Plant Biol. 2008 Sep 16;8:93. doi: 10.1186/1471-2229-8-93 (PMC2564936; doi:10.1186/1471-2229-8-93)
Supplement: Additional file 3 — Detail putative targets of high-copy small RNAs (> 5 copies) in developing ovules of cotton. [file 1471-2229-8-93-S3.doc]

| **Table S2. Putative targets of high-copy small RNAs (>5 copies) in developing ovules of cotton** | | | | | |
| --- | --- | --- | --- | --- | --- |
| **sRNA name** | **Sequence 5' – 3'** | **L (nt)** | **NC** | **Putative target protein ID** | **Putative taget protein names** |
| **0 dpa** |  |  |  |  |  |
| Gh_sRNA_0dpa22 | ACUAGCCCUAAGUGGAGGUCCAUU | 24 | 14 | At1g32960.1 (3); At5g48930.1 (3) | transferase family protein (similar to anthranilate N-hydroxycinnamoyl/benzoyltransferase); subtilase family protein |
| Gh_sRNA_0dpa03 | AGCGAAAAAAUGGUACCCAUGCGAU | 25 | 8 | At1g03610.1 (3) | expressed protein |
| Gh_sRNA_0dpa39 | AAGAAAAUCAGCCUAAGAUGGUCC | 24 | 7 | At2g15490.1 (2.5); At5g19370.1 (3); At5g37030.1 (3); At5g37150.1 (3); At2g46270.1 (3); At2g46270.2 (3); At2g46915.1 (3); At4g26140.1 (3); At4g26140.2 (3) | beta-galactosidase (putative lactase)(2); rhodanese-like domain-containing protein / PPIC-type PPIASE domain-containing protein; G-box binding factor 3 (GBF3)(2); tRNA-splicing endonuclease positive effector-related(2); UDP-glucoronosyl/UDP-glucosyl transferase family protein; expressed protein |
| Gh_sRNA_0dpa102 | CCAUCGGACUUGACCGAAGAAG | 22 | 7 | At5g15790.1 (1.5); At5g15790.2 (1.5); At1g72870.1 (2.5); At1g22090.1 (3); At4g32440.1 (3); At5g44950.1 (3) | zinc finger (C3HC4-type RING finger) family protein; disease resistance protein (TIR-NBS class); F-box family protein; agenet domain-containing protein |
| Gh_sRNA_0dpa08 | GUUGAACGAGGUUAAGAACUAAUU | 24 | 5 | At5g20160.1 (1.5); At5g20160.2 (1.5); At5g17990.1 (2); At1g02810.1 (3); At1g05150.1 (3); At1g13090.1 (3); At3g02260.1 (3); At5g17630.1 (3); At2g32275.1 (3); At3g27340.1 (3); At4g33460.1 (3); At4g12600.1 (3) | anthranilate phosphoribosyltransferase; pectinesterase family protein; expressed protein(2); ribosomal protein L7Ae/L30e/S12e/Gadd45 family protein(3); glucose-6-phosphate/phosphate translocator; cytochrome P450 71B28; calcium-binding EF hand family protein; auxin transport protein (BIG) nearly identical to auxin transport protein; ABC transporter family protein |
| Gh_sRNA_0dpa24 | AGACCGAUAAUCCUGAUGUGGUCC | 24 | 5 | At4g25960.1 (3) | multidrug resistance P-glycoprotein |
| Gh_sRNA_0dpa42 | AUCUCUGAGGAAAUGGUCAAAAGA | 24 | 5 | At2g17210.1 (2); At3g57200.1 (3); At2g26650.1 (3); At4g39440.1 (3); At4g15093.1 (3); At4g24120.1 (3); At5g02180.1 (3); At5g51900.1 (3); At3g54520.1 (3) | expressed protein expression supported by MPSS; pentatricopeptide (PPR) repeat-containing protein; amino acid transporter family protein; hypothetical protein(2); transporter (putative similar to iron-phytosiderophore transporter protein yellow stripe 1); hypothetical protein; cytochrome P450 family; potassium channel protein 1 (AKT1); catalytic LigB subunit of aromatic ring-opening dioxygenase family |
| Gh_sRNA_0dpa81 | CGAUGAAUCUGCCCUCGCCCGCU | 23 | 5 | At1g09795.1 (2) | ATP phosphoribosyl transferase 2 (ATP-PRT2) |
| Gh_sRNA_0dpa82 | UUCACCCUUGAGUCUCGGAGCAA | 23 | 5 | At5g28140.1 (3); At1g11080.1 (3); At1g68580.1 (3); At1g68580.2 (3) | agenet domain-containing protein / bromo-adjacent homology (BAH) domain-containing protein; serine carboxypeptidase S10 family protein; hypothetical protein; agenet domain-containing protein / bromo-adjacent homology (BAH) domain-containing protein |
| **1 dpa** |  |  |  |  |  |
| Gh_sRNA_1dpa26 | AGUAUAUUUCGAUAGUUAAAGUGC | 24 | 12 | At1g68100.1 (2.5); At5g09620.1 (2.5); At5g54870.1 (3); At5g58110.1 (3) | octicosapeptide/Phox/Bem1p (PB1) domain-containing protein; expressed protein(2); IAA-alanine resistance protein 1 |
| Gh_sRNA_1dpa17 | AAAAAAUUAACCGAACCGAAAUUU | 24 | 11 | At5g52790.1 (2.5); At1g14790.1 (2.5); At1g29690.1 (2.5); At3g29763.1 (3); At2g02780.1 (3); At4g01670.1 (3); At2g42130.1 (3); At2g42130.2 (3); At2g42130.3 (3); At2g42130.4 (3); At2g42130.5 (3) | expressed protein(7); hypothetical protein; RNA-dependent RNA polymerase; CBS domain-containing protein-related |
| Gh_sRNA_1dpa12 | AAACACACCGCCCAUCCAAACUCA | 24 | 9 | At1g10750.1 (3); At1g65180.1 (3); At5g22020.1 (3) | expressed protein; strictosidine synthase family protein; DC1 domain-containing protein |
| Gh_sRNA_1dpa67 | UUAGCCUUAAAAAACGAUCAUGC | 23 | 9 | At2g33655.1 (3) | F-box family protein |
| Gh_sRNA_1dpa24 | AUUGUUCUUAGUUAUGUGUGCUUA | 24 | 8 | At1g30410.1 (2.5); At1g35910.1 (3); At5g41250.1 (3); At1g65370.1 (3); At5g46380.1 (3); At3g12290.1 (3); At5g02740.1 (3); At5g02740.2 (3); At5g52975.1 (3); At3g25690.1 (3) | expressed protein(2); trehalose-6-phosphate phosphatase; tetrahydrofolate dehydrogenase/cyclohydrolase (putative similar to C-1-tetrahydrofolate synthase, cytoplasmic (C1-THF synthase)); meprin and TRAF homology domain-containing protein / MATH domain-containing protein; hypothetical protein; exostosin family protein; ATP-binding cassette transport protein (putative similar to MgATP-energized glutathione S-conjugate pump) |
| Gh_sRNA_1dpa23 | AUUACUAUAAAUCGAAGUGUCGAA | 24 | 7 | At1g80740.1 (2.5 y); At1g18670.1 (3); At3g07650.1 (3); At3g07650.2 (3) | chromomethylase 1 (CMT1); protein kinase family protein; zinc finger (B-box type) family protein(2) |
| Gh_sRNA_1dpa16 | AAACCUUACCUGAACGGAGCGCAA | 24 | 6 | TC37780 (3); TC38238 (3) | similar to PRP8 protein; weakly similar to KH domain RNA binding protein QKI-7B |
| Gh_sRNA_1dpa18 | CAACCUGAUUUUUUAUUCUAGAAC | 24 | 6 | At3g16180.1 (2); At5g61430.1 (2.5); At1g57906.1 (3); At1g48760.1 (3); At1g48760.2 (3); At4g02720.1 (3); At4g24265.1 (3); At2g37670.1 (3); At5g28320.1 (3); At5g28400.1 (3) | expressed protein(4); hypothetical protein; proton-dependent oligopeptide transport (POT) family protein; no apical meristem (NAM) family protein; delta-adaptin (putative similar to Adapter-related protein complex 3 delta 1 subunit (Delta-adaptin 3))(2); WD-40 repeat family protein |
| Gh_sRNA_1dpa30 | AAUCGUCGAAUCUAAAUGUGUGAC | 24 | 6 | At1g26650.1 (2.5 ); At3g56330.1 (2.5 ); At1g06710.1 (2.5 ); At2g31650.1 (2.5 ); At4g26580.1 (2.5 ); At5g16960.1 (2.5 ); At3g11420.1 (3 ); At1g33700.1 (3 ); At1g66110.1 (3 ); At2g24930.1 (3 ); At5g22760.1 (3 ); | hypothetical protein; PHD finger family protein; fringe-related protein; expressed protein(2); NADP-dependent oxidoreductase; Ulp1 protease family protein; trithorax 1 (ATX-1) (TRX1); N2,N2-dimethylguanosine tRNA methyltransferase family protein; zinc finger (C3HC4-type RING finger) family protein; pentatricopeptide (PPR) repeat-containing protein |
| Gh_sRNA_1dpa68 | AUCAGUGCCAUAAAUUGAAGAAG | 23 | 6 | At2g04100.1 (2.5); At2g34070.1 (2.5 y); At2g45540.1 (2.5); At5g40480.1 (2.5); At5g19620.1 (3); At1g51590.1 (3); At2g25710.1 (3); At2g25710.2 (3); At2g31110.1 (3 y); At3g05140.1 (3); At5g06230.1 (3 y); At5g06230.2 (3 y); At1g26170.1 (3); At3g08760.1 (3); At4g14300.1 (3); | WD-40 repeat family protein / beige-related; protein kinase family protein(2); expressed protein(5); expression supported by MPSS; importin beta-2 subunit family; outer membrane OMP85 family protein; MATE efflux family protein (similar to ripening regulated protein DDTFR18); mannosyl-oligosaccharide 1,2-alpha-mannosidase; holocarboxylase synthetase 1 (HCS1)(2); heterogeneous nuclear ribonucleoprotein |
| **2 dpa** |  |  |  |  |  |
| Gh_sRNA_2dpa111 | UGAAACUUUAGCCGUGGUUGGA | 22 | 22 | At2g39930.1 (3); At1g21323.1 (3); At1g36390.1 (3); At1g36390.2 (3); At4g21410.1 (3 y); At4g29060.1 (3); At4g03550.1 (3) | protein kinase family protein; isoamylase (putative starch debranching enzyme); co-chaperone grpE family protein(2); glycosyl transferase family 48 protein; hypothetical protein;  elongation factor Ts family protein |
| Gh_sRNA_2dpa29 | AAGGAACACCACUCGAGACCCCAA | 24 | 18 | At4g28350.1 (3); At2g19710.1 (3); At4g29450.1 (3); At1g61740.1 (3) | lectin protein kinase family protein; expressed protein(2); leucine-rich repeat protein kinase (putative similar to light repressible receptor protein kinase) |
| Gh_sRNA_2dpa119 | AGAAUCCUGAUGAUGCUGCAG | 21 | 13 | At4g36920.1 (0 y); At5g60120.1 (0 y); At2g28550.1 (1 y); At2g28550.2 (1 y); At5g67180.1 (1 y); At2g39250.1 (1 y); At2g28056.1 (2.5); At3g12590.1 (2.5); At4g29430.1 (2.5); At4g37030.1 (3) | floral homeotic protein APETALA2 (AP2); 40S ribosomal protein S15A (RPS15aE); AP2 domain-containing transcription factor (putative Similar to Floral homeotic protein APETALA2 protein)(5); expressed protein(2); hypothetical protein |
| Gh_sRNA_2dpa28 | UAUUCAGAAAAUGCUCUAUCCACC | 24 | 12 | At4g08170.1 (2); At4g08170.2 (2); At5g67610.1 (2); At1g20710.1 (2.5); At3g43880.1 (2.5); At5g37150.1 (3); At1g20700.1 (3); At1g28020.1 (3 y); At5g18410.1 (3); At5g18410.2 (3) | expressed protein (similar to p53 inducible protein)(2); homeobox-leucine zipper family protein (similarity to homeodomain protein PALE-2)(2); hypothetical protein p97; inositol 1,3,4-trisphosphate 5/6-kinase family protein(2); tRNA-splicing endonuclease positive effector-related; expressed protein; pentatricopeptide (PPR) repeat-containing protein |
| Gh_sRNA_2dpa27 | ACAAUUUGUCCCCUACCUUGUUGA | 24 | 9 | At3g42200.1 (3) | hypothetical protein |
| Gh_sRNA_2dpa38 | AACCUGCAUCUCCACCUUAUUAUU | 24 | 8 | TC31880 (2.5); TC40987 (3); TC39141 (3) | similar to DNA binding zinc finger protein (Pspzf) (Fragment); weakly similar to UP|Q9LPJ0; weakly similar to Cyclin-like protein |
| Gh_sRNA_2dpa107 | ACUUCAUUGUAGUCAAUCCCCU | 22 | 7 | At3g60400.1 (2); At5g18750.1 (2.5 y); At5g46510.1 (3 y); At1g61190.1 (3 y); At5g64630.1 (3); At5g64630.2 (3); At5g64630.3 (3); At2g32990.1 (3); At2g39100.1 (3); At5g37760.1 (3 y); At5g45660.1 (3) | zinc finger (C3HC4-type RING finger) family protein; DNAJ heat shock N-terminal domain-containing protein(2); transducin family protein / WD-40 repeat family protein(3); glycosyl hydrolase family 9 protein (similar to endo-beta-1,4-glucanase); disease resistance protein (CC-NBS-LRR class)(2); mitochondrial transcription termination factor-related / mTERF-related; expressed protein |
| Gh_sRNA_2dpa120 | GAGUUAGACUUUGCAGACAUC | 21 | 6 | At5g27110.1 (2); At5g38565.1 (2); At1g77310.1 (3); At3g55660.1 (3); At1g53790.1 (3); At2g01820.1 (3); At3g12560.1 (3); At5g37130.1 (3) | tetratricopeptide repeat (TPR)-containing protein; F-box family protein(2); expressed protein; telomeric DNA-binding protein; wound-responsive protein; pentatricopeptide (PPR) repeat-containing protein; leucine-rich repeat protein kinase (putative similar to protein kinase TMK1) |
| Gh_sRNA_2dpa09 | AUCGUUUCGAGAUGAGUCAAACUA | 24 | 5 | At1g58250.1 (2); At3g06620.1 (2.5 y); At3g51530.1 (3 y); At1g50575.1 (3); At3g20820.1 (3 y); At4g33700.1 (3); At5g18230.1 (3); At2g46870.1 (3); At3g05545.1 (3); At5g15540.1 (3); At5g56760.1 (3) | transcription factor, putative / zinc finger (C3HC4 type RING finger) family protein (similar to VIP2 protein); expressed protein; transcription regulator NOT2/NOT3/NOT5 family protein; serine O-acetyltransferase (SAT-52); F-box family protein; CBS domain-containing protein; SABRE; lysine decarboxylase family protein; leucine-rich repeat family protein; protein kinase family protein; DNA-binding protein |
| **3 dpa** |  |  |  |  |  |
| Gh_sRNA_3dpa16 | GAAAUGAGGUUCGUGGGAUUGUGC | 24 | 90 | At3g06270.1 (1.5); At1g17690.1 (2.5); At5g45390.1 (3); At1g26560.1 (3); At1g66980.1 (3 y); At5g24280.1 (3) | protein kinase family protein / glycerophosphoryl diester phosphodiesterase family protein (similar to leaf rust resistance kinase Lr10); protein phosphatase 2C; ATP-dependent Clp protease; expressed protein; expressed protein expression supported by MPSS; glycosyl hydrolase family 1 protein |
| Gh_sRNA_3dpa25 | CACAUGCCAACCGAUUGAUCGC | 22 | 31 | At5g14020.1 (2.5); At5g37100.1 (2.5) | replication protein-related; expressed protein |
| Gh_sRNA_3dpa17 | AAAGACUGAAGGUAAGGAGAGUGC | 24 | 27 | At1g34430.1 (2.5); At1g80920.1 (2.5); At5g19875.1 (2.5); At4g23590.1 (2.5); At3g50570.1 (2.5); At1g04620.1 (3); At3g42722.1 (3); At1g51730.1 (3); At1g73430.1 (3); At3g12320.1 (3); At5g24280.1 (3); At1g62480.1 (3); At1g69420.1 (3); At2g40420.1 (3); At5g04080.1 (3) | expressed protein(3); coenzyme F420 hydrogenase family / dehydrogenase, beta subunit family; sec34-like family protein; dihydrolipoamide S-acetyltransferase; DNAJ heat shock N-terminal domain-containing protein (similar to Chaperone protein dnaJ); expressed protein expression supported by MPSS; zinc finger (DHHC type) family protein; vacuolar calcium-binding protein-related; amino acid transporter family protein (similar to neuronal glutamine transporter); aminotransferase class I and II family protein (similar to nicotianamine aminotransferase); hydroxyproline-rich glycoprotein family protein; RWD domain-containing protein (similar to RING finger protein 25); F-box family protein |
| Gh_sRNA_3dpa9 | ACUGGCGCUGAGUGGUGUGUAAGA | 24 | 21 | At3g57150.1 (2.5); At5g59950.3 (3) | dyskerin, putative / nucleolar protein NAP57; RNA and export factor-binding protein |
| Gh_sRNA_3dpa18 | CGCCCUCUUUAGUUUGGUGAAUGC | 24 | 21 | At5g58370.1 (3); At5g58370.2 (3) | expressed protein(2) |
| Gh_sRNA_3dpa11 | GAGCCUCCCCAAAUCAAAUGGUAC | 24 | 12 | At1g58807.1 (3); At1g59124.1 (3); At1g22840.1 (3); At5g24970.1 (3) | ABC1 family protein; disease resistance protein (CC-NBS-LRR class)(2); cytochrome c |
| Gh_sRNA_3dpa3 | AGGUCAUGAGAGGCCCACAUGAGC | 24 | 11 | At2g31740.1 (2); At5g53510.1 (3) | oligopeptide transporter OPT family protein (similar to Sexual differentiation process protein isp4); expressed protein |
| Gh_sRNA_3dpa13 | AUGGAUAGGGUUCGAGCUGGGUCU | 24 | 11 | At5g04530.1 (3); At2g17265.1 (3); At1g58025.1 (3); | DNA-binding bromodomain-containing protein; homoserine kinase (HSK); beta-ketoacyl-CoA synthase family protein KCS1 fatty acid elongase 3-ketoacyl-CoA synthase 1 |
| Gh_sRNA_3dpa26 | AGAAUCCUGAUGAUGCUGCAG | 21 | 9 | At4g36920.1 (0 y); At5g60120.1 (0 y); At2g28550.1 (1 y); At2g28550.2 (1 y); At5g67180.1 (1 y); At2g39250.1 (1 y); At2g28056.1 (2.5); At3g12590.1 (2.5);  At4g29430.1 (2.5); At4g37030.1 (3) | floral homeotic protein APETALA2 (AP2); 40S ribosomal protein S15A (RPS15aE); AP2 domain-containing transcription factor (putative similar to Floral homeotic protein APETALA2 protein)(3); expressed protein(2); AP2 domain-containing transcription factor RAP2.7(2); hypothetical protein |
| Gh_sRNA_3dpa28 | CCCAGUCCCGAACCCGUCGGC | 21 | 9 | None | None |
| Gh_sRNA_3dpa8 | AUGACCCGGUUGAUGGAUGAUACC | 24 | 8 | At4g27560.1 (3); At4g27570.1 (3); At5g27560.1 (3); At5g46780.1 (3); At5g46780.2 (3); At1g54490.1 (3);At4g21350.1 (3) | U-box domain-containing protein (similar to immediate-early fungal elicitor protein CMPG1); glycosyltransferase family protein; 5'-3' exoribonuclease (XRN4); glycosyltransferase family protein; expressed protein (hypothetical protein slr1702); VQ motif-containing protein(2) |
| Gh_sRNA_3dpa5 | AAGAGAACGUGGGUUAGAGUGUGC | 24 | 7 | At1g02570.1 (2); At1g04050.1 (2.5); At3g21690.1 (2.5); At2g35710.1 (3); At2g35710.2 (3); At1g51480.1 (3); At4g10550.1 (3); At1g17600.1 (3); At1g51570.1 (3); At3g09120.1 (3) | expressed protein; SET domain-containing protein / suppressor of variegation related 1 (SUVR1) (identical to suppressor of variegation related 1); disease resistance protein (TIR-NBS-LRR class); MATE efflux family protein (similar to ripening regulated protein DDTFR18); glycogenin glucosyltransferase (glycogenin)-related(2); disease resistance protein (CC-NBS-LRR class); subtilase family protein (similarity to subtilisin-like protease AIR3) GI:4218991 from [Arabidopsis thaliana] . |
| Gh_sRNA_3dpa6 | AAUUUUGGGUGUGACGAGGGUGAU | 24 | 7 | At1g06000.1 (2); At4g13640.1 (2.5); At5g46260.1 (2.5); At1g01300.1 (3); At3g56820.1 (3); At1g29860.1 (3) | myb family transcription factor; disease resistance protein (TIR-NBS-LRR class); WRKY family transcription factor (similar to DNA-binding protein 2); expressed protein; aspartyl protease family protein; UDP-glucoronosyl/UDP-glucosyl transferase family protein |
| Gh_sRNA_3dpa10 | AUCGUCGGUGCAUAAGUCUCGUGC | 24 | 7 | At5g09210.1 (2.5); At4g17430.1 (3); At1g49120.1 (3) | hypothetical protein; AP2 domain-containing transcription factor; expressed protein |
| Gh_sRNA_3dpa27 | UUGUGUUAUAGAGAAUGGAGC | 21 | 7 | At1g05580.1 (3); At3g57480.1 (3) | zinc finger (C2H2 type, AN1-like) family protein; cation/hydrogen exchanger (putative (CHX23) monovalent cation:proton antiporter family 2 (CPA2) member) |
| Gh_sRNA_3dpa12 | AAGAGGCUGUGUGGCUCACUGUGC | 24 | 6 | TC39177 (3) | similar to UP|Q8VYI1 |
| Gh_sRNA_3dpa30 | GGAUGAACAGGAUGAGUGU | 19 | 6 | None | None |
| Gh_sRNA_3dpa4 | UUUUUCACUGUCCAAGGUAAGCCU | 24 | 5 | At1g43245.1 (2); At5g14510.1 (2.5); At1g65150.1 (2.5); At1g65150.2 (2.5); At4g13430.1 (2.5); At2g03140.1 (2.5); At5g50360.1 (2.5); At1g65050.1 (3); At2g25050.1 (3 y); At3g09790.1 (3); At4g23440.1 (3 y); At1g23180.1 (3 y); At2g35330.1 (3); At3g07960.1 (3); At4g19510.1 (3 y); At4g14980.1 (3 y); At1g27710.1 (3); At4g38600.1 (3 y); At4g38600.2 (3 y) | armadillo/beta-catenin repeat family protein(2); CAAX amino terminal protease family protein; HECT-domain-containing protein / ubiquitin-transferase family protein (similar to Thyroid receptor interacting protein 12 (TRIP12))(3); meprin and TRAF homology domain-containing protein / MATH domain-containing protein (similar to ubiquitin-specific protease 12)(3); DC1 domain-containing protein; glycine-rich protein; zinc finger (C3HC4-type RING finger) protein-related; expressed protein(3); aconitase family protein / aconitate hydratase family protein; formin homology 2 domain-containing protein / FH2 domain-containing protein; phosphatidylinositol-4-phosphate 5-kinase family protein; polyubiquitin (UBQ8) identical; disease resistance protein (TIR-NBS-LRR class) |
| Gh_sRNA_3dpa7 | GAAUUAAGGGUCCGUUUGAUUGCU | 24 | 5 | At1g07990.1 (2); At2g02710.2 (2); At3g46690.1 (3); At5g03180.1 (3) | PAC motif-containing protein (similar to nonphototropic hypocotyl 1); zinc finger (C3HC4-type RING finger) family protein; UDP-glucoronosyl/UDP-glucosyl transferase family protein; SIT4 phosphatase-associated family protein |
| **4 dpa** |  |  |  |  |  |
| Gh_sRNA_4dpa14 | AUGGGUUGAGAGAUGGGUUAUGAG | 24 | 27 | At4g13050.1 (2); At4g36180.1 (2.5 y); At5g18420.1 (2.5); At5g18420.2 (2.5); At5g18420.3 (2.5); At5g24350.1 (3); At3g33530.1 (3); At1g78000.1 (3); At2g31040.1 (3); At3g47110.1 (3 y); At1g52510.1 (3); At4g15070.1 (3); At5g48470.1 (3); At2g15900.1 (3); At4g04390.1 (3); At4g33720.1 (3); At1g71420.1 (3); | ATP synthase protein I -related; leucine-rich repeat transmembrane protein kinase (putative protein kinase Xa21 receptor type precursor); acyl-[acyl carrier protein] thioesterase; leucine-rich repeat family protein; expressed protein(5); transducin family protein / WD-40 repeat family protein (similar to WD-repeat protein 11); phox (PX) domain-containing protein; sulfate transporter (Sultr1&2); pentatricopeptide (PPR) repeat-containing protein; DC1 domain-containing protein; pathogenesis-related protein; Ulp1 protease family protein; hydrolase, alpha/beta fold family protein |
| Gh_sRNA_4dpa5 | AUAAAAGGUCGUGAGUUUGAGUGC | 24 | 20 | At5g05870.1 (2.5); At1g80240.1 (3); At2g21050.1 (3); At1g68320.1 (3); At2g24630.1 (3); At4g03090.1 (3); At1g03040.1 (3); At1g20090.1 (3); At2g41380.1 (3); At3g02260.1 (3); At4g13030.1 (3); At4g13030.2 (3) | expressed protein(4); Rac-like GTP-binding protein (ARAC4) / Rho-like GTP-binding protein (ROP2); glycosyl transferase family 2 protein (similar to cellulose synthase); embryo-abundant protein-related; basic helix-loop-helix (bHLH) family protein component of the pyruvate dehydrogenase complex E3; amino acid permease (putative similar to AUX1); UDP-glucoronosyl/UDP-glucosyl transferase family protein; myb family transcription factor (MYB62); auxin transport protein (BIG) |
| Gh_sRNA_4dpa30 | GCACGUCUGCCUGGGUGUCACGC | 23 | 16 | None | None |
| Gh_sRNA_4dpa12 | AAAUCGUGCCCUAACGUAUUGAGU | 24 | 15 | TC38237 (2); TC38974 (2); TC28113 (3) | similar to S-locus protein 3; similar to UP|Q8S9L4; Polygalacturonase-inhibiting protein precursor |
| Gh_sRNA_4dpa15 | AAUGUUGGGUCCUAACUUACUGGU | 24 | 15 | At1g67120.1 (2); At1g74130.1 (3); At1g74130.2 (3) | rhomboid family protein; rhomboid family protein; midasin-related |
| Gh_sRNA_4dpa34 | GGCAAUCAUCCUUGGUUAAGC | 21 | 15 | At3g44340.1 (1.5); At2g19120.1 (3); At4g28470.1 (3); At4g10760.1 (3 y) | tRNA-splicing endonuclease positive effector-related (similar to Endonuclease sen1); methyltransferase MT-A70 (putative similar to (N6-adenosine)-methyltransferase); 26S proteasome regulatory subunit; sec23/sec24 transport family protein |
| Gh_sRNA_4dpa16 | AUAAUGGUCCGAACAAAGGGGUUG | 24 | 13 | At5g16920.1 (2.5); At1g22882.1 (3); At2g25540.1 (3); At5g48205.1 (3) | hypothetical protein; expressed protein(2); cellulose synthase, catalytic subunit (similar to cellulose synthase-1) |
| Gh_sRNA_4dpa2 | AGUGUCACGGAACAAAUGUCUUGAU | 25 | 12 | At1g30475.1 (2.5); At1g62290.1 (3) | expressed protein; aspartyl protease family protein |
| Gh_sRNA_4dpa9 | AAAUGAUAGGCUUGCCCGGGUGGU | 24 | 11 | At5g11110.1 (2.5); At2g44590.1 (3); At2g44590.2 (3); At2g44590.3 (3); At3g46240.1 (3) | dynamin-like protein D (DL1D)(3); protein kinase-related (similar to light repressible receptor protein kinase) |
| Gh_sRNA_4dpa11 | AGGUGCAAGAUUCCGAUGAAGAUG | 24 | 9 | At2g34380.1 (2.5); At3g59410.1 (2.5); At5g16580.1 (2.5); At1g49950.1 (2.5); At5g01950.1 (2.5 y); At5g44700.1 (3 y); At1g62390.1 ( 3 y); At1g32170.1 (3); At3g07210.1 (3); At3g26190.1 (3); At3g26300.1 (3); At4g39470.1 (3 y); At5g05480.1 (3); At1g28390.1 (3) | expressed protein (3); chloroplast lumen common family protein; cytochrome P450 family protein; DNA-binding protein (similar to PcMYB1)(2); leucine-rich repeat transmembrane protein kinase(2); cytochrome P450 71B21; protein kinase family protein(2); xyloglucan:xyloglucosyl transferase; octicosapeptide/Phox/Bem1p (PB1) domain-containing protein / tetratricopeptide repeat (TPR)-containing protein; glycosyl hydrolase family 1 protein |
| Gh_sRNA_4dpa1 | UGUUAACUCCCUUGAUGGAUCCAAC | 25 | 8 | At2g31300.1 (2); At2g30910.1 (2.5); At2g30910.2 (2.5);At5g64370.1 (3); At1g24706.1 (3); At4g37590.1 (3) | phototropic-responsive NPH3 family protein; expressed protein; transducin family protein / WD-40 repeat family protein; beta-ureidopropionase (putative beta-alanine synthase); transducin family protein / WD-40 repeat family protein; transducin family protein / WD-40 repeat family protein |
| Gh_sRNA_4dpa6 | AGGGUGAGCGUUUGAUUGAGUUGA | 24 | 8 | At3g15690.1 (2.5); At5g08390.1 (2.5); At3g55000.1 (3); At4g16770.1 (3); At4g35790.3 (3); At3g02290.2 (3) | phospholipase D delta / PLD delta (PLDDELTA)(3); transducin family protein / WD-40 repeat family protein (similar to katanin p80 subunit); biotin carboxyl carrier protein of acetyl-CoA carboxylase-related; tonneau family protein (similar to tonneau 1b)(2); oxidoreductase, 2OG-Fe(II) oxygenase family protein; zinc finger (C3HC4-type RING finger) family protein |
| Gh_sRNA_4dpa7 | CAAACUGGGCACGUACCGAACAUC | 24 | 8 | TC32342 (3); TC32345 (3) | similar to GB|AAO23588.1; similar to GB|AAS99687.1 |
| Gh_sRNA_4dpa10 | AAAAUCUUACUUGAGGCUCGUUCC | 24 | 8 | At2g29560.1 (2.5); At5g17760.2 (2.5); At3g62670.1 (3); At1g51620.1 (3) | protein kinase family protein; enolase; two-component responsive regulator family protein; AAA-type ATPase family protein |
| Gh_sRNA_4dpa18 | AAAGCUUCGACGAUAACACGGUGG | 24 | 7 | At1g74390.1 (2.5); At4g18700.1 (3); At5g42390.1 (3); At2g42120.1 (3); At2g42120.2 (3) | exonuclease family protein; CBL-interacting protein kinase 12 (CIPK12); DNA polymerase delta small subunit-related; metalloendopeptidase (identical to chloroplast processing enzyme metalloendopeptidase) |
| Gh_sRNA_4dpa19 | AAACAUGCCUGAAUCACUGUGAAA | 24 | 7 | At5g65810.1 (2.5); At1g08610.1 (3); At4g38900.1 (3); At4g38900.2 (3); | bZIP protein vsf-1 protein(2); pentatricopeptide (PPR) repeat-containing protein; expressed protein |
| Gh_sRNA_4dpa8 | CUCAAAUUAACUCGAUACCGACUC | 24 | 6 | At5g51040.1 (3) | expressed protein |
| **5 dpa** |  |  |  |  |  |
| Gh_sRNA_5dpa28 | AUAUCUAGAGUGAUCGCAAUUACC | 24 | 53 | At4g16860.1 (2); At4g16890.1 (2); At4g16900.1 (2); At4g16920.1 (2); At4g16940.1 (2); At4g16960.1 (2); At1g75810.1 (3); At2g46340.1 (3 y); At4g16950.1 (3); At3g16560.1 (3) | disease resistance protein (TIR-NBS-LRR class)(8); protein phosphatase 2C-related / PP2C-related contains protein phosphatase 2C domain; phytochrome A supressor spa1 (SPA1); expressed protein |
| Gh_sRNA_5dpa13 | AAUUUUAUUCAUAAUGACCAAGAC | 24 | 23 | At1g79390.1 (2.5); At2g03760.1 (3); At5g28300.1 (3); At2g40270.1 (3 y); At2g40270.2 (3 y); At1g04500.1 (3); At1g52140.1 (3); At1g61540.1 (3); At1g74450.1 (3); At4g19440.1 (3 y); At5g01960.1 (3) | expressed protein(3); kelch repeat-containing F-box family protein; pentatricopeptide (PPR) repeat-containing protein; protein kinase family protein; steroid sulfotransferase; DNA-binding protein (putative similar to GT-2 factor); zinc finger (C3HC4-type RING finger) family protein; zinc finger CONSTANS-related (similar to Zinc finger protein constans-like 15) |
| Gh_sRNA_5dpa08 | ACAACUUAGUGACUUAAAUGAGAA | 24 | 16 | At2g31500.1 (2); At3g13080.3 (3); At3g13080.4 (3) | ABC transporter family protein (identical to MRP-like ABC transporter)(2); calcium-dependent protein kinase |
| Gh_sRNA_5dpa07 | AUGAACUUACCUCGAUACCACAAA | 24 | 13 | At5g51020.1 (2 5); At3g53010.1 (2.5); At5g18040.1 (3); At4g24265.1 (3) | expressed protein(4) |
| Gh_sRNA_5dpa32 | AAUGGUGUCUGACAUUUAUGACA | 23 | 11 | At3g12810.1 (3 y); At3g28650.1 (3); At3g33520.1 (3); At2g33240.1 (3); At4g10845.1 (3); At5g45370.1 (3); At5g45370.2 (3); At5g45370.3 (3); At2g20530.1 (3); At5g18740.1 (3); At5g23860.1 (3) | expressed protein; nodulin-related / integral membrane family protein(3); prohibitin; hypothetical protein; actin-related protein 6 (ARP6); DC1 domain-containing protein; SNF2 domain-containing protein / helicase domain-containing protein (similar to transcriptional activator SRCAP); myosin; tubulin beta-8 chain (TUB8) (TUBB8) |
| Gh_sRNA_5dpa35 | AAAGUGUGUAAUUGAAGUAUCGG | 23 | 11 | At2g18130.1 (2); At3g46120.1 (2); At1g17540.1 (2.5); At3g20480.1 (3); At3g19810.1 (3); At1g75380.1 (3); At1g75380.2 (3); At1g75380.3 (3) | calcineurin-like phosphoesterase family protein; tetraacyldisaccharide 4'-kinase family protein; expressed protein; protein kinase-related (similar to serine/threonine protein kinase Fen); purple acid phosphatase (PAP11); wound-responsive protein-related (similar to wound inducive gene)(3) |
| Gh_sRNA_5dpa44 | GAAGCGCUGGAGGGUAGAGUGG | 22 | 10 | At2g25340.1 (3); At2g32550.1 (3); At1g12820.1 (3); At1g14730.1 (3); At3g22660.1 (3); At4g01380.1 (3) | cytochrome B561 family; transport inhibitor response protein (putative E3 ubiquitin ligase SCF complex F-box subunit); synaptobrevin family protein; rRNA processing protein-related; plastocyanin-like domain-containing protein |
| Gh_sRNA_5dpa06 | UUUGUCAAGUUCAGAGACCAAAAG | 24 | 9 | At2g30800.1 (1.5); At4g33760.1 (2); At5g51830.1 (2.5); At1g57790.1 (2.5); At5g35200.1 (2.5); At1g32310.1 (3); At2g05170.1 (3); At5g08580.1 (3); At1g34140.1 (3); At1g48410.1 (3); At1g48410.2 (3); At2g36530.1 (3); At3g10440.1 (3); At3g53880.1 (3); At1g22550.1 (3); At4g25310.1 (3); At4g13750.1 (3); At4g32360.1 (3) | calcium-binding EF hand family protein; oxidoreductase, 2OG-Fe(II) oxygenase family protein (similar to flavonol synthase); NADP adrenodoxin-like ferredoxin reductase; hypothetical protein; pfkB-type carbohydrate kinase family protein; tRNA synthetase class II (D, K and N) family protein (similar to Aspartyl-tRNA synthetase); F-box family protein; polyadenylate-binding protein (similar to polyadenylate-binding protein (poly(A)-binding protein)); expressed protein(2); vacuolar protein sorting 11 family protein / VPS11 family protein; proton-dependent oligopeptide transport (POT) family protein; epsin N-terminal homology (ENTH) domain-containing protein (similar to clathrin assembly protein); argonaute protein (AGO1)(2); DEIH-box RNA/DNA helicase; aldo/keto reductase family protein (similar to chalcone reductase, aldose reductase ALDRXV4); enolase identical |
| Gh_sRNA_5dpa10 | AUAUUCGAGAAACGUGCGGUGACU | 24 | 9 | At2g04840.1 (2.5); At5g03530.1 (2.5) | Ras-related GTP-binding family protein; F-box family protein |
| Gh_sRNA_5dpa33 | UAAACCUAGAACCUCACACACAC | 23 | 8 | At1g14380.1 (3); At1g14380.2 (3); At2g20130.1 (3); At4g03115.1 (3); At4g28530.1 (3); At3g51560.1 (3) | calmodulin-binding family protein(2); no apical meristem (NAM) family protein; disease resistance protein (TIR-NBS-LRR class); expressed protein; mitochondrial substrate carrier family |
| Gh_sRNA_5dpa14 | AACACUCCCGGAUUCGGCCAUAAU | 24 | 7 | At4g12750.1 (3) | expressed protein |
| Gh_sRNA_5dpa22 | AUACUCCCUUUGCUAUGCACUUCA | 24 | 7 | At1g48570.1 (3) | zinc finger (Ran-binding) family protein |
| Gh_sRNA_5dpa34 | GUUUUAUAUCAGAUCCUUGAACA | 23 | 7 | At5g06930.1 (2.5); At5g43920.1 (2.5); At1g72730.1 (3); At3g54380.1 (3); At3g54380.2 (3); At3g50380.1 (3); At5g52850.1 (3 y); At1g22630.1 (3); At3g44110.1 (3); At3g44110.2 (3) | DNAJ heat shock protein; expressed protein(3); SAC3/GANP family protein(2); DNAJ heat shock protein (identical to AtJ3); transducin family protein / WD-40 repeat family protein contains 7 WD-40 repeats (PF00400) (similar to will die slowly protein (WDS)); pentatricopeptide (PPR) repeat-containing protein; eukaryotic translation initiation factor 4A |
| Gh_sRNA_5dpa04 | ACUCCCACUUACACUUGGUGUGCA | 24 | 6 | At5g33410.1 (2.5); At5g24710.1 (2.5); At4g14030.1 (3); At5g11710.1 (3); At5g60170.1 (3) | RNA recognition motif (RRM)-containing protein; WD-40 repeat family protein; epsin N-terminal homology (ENTH) domain-containing protein / clathrin assembly protein-related; selenium-binding protein; replication protein-related |
| Gh_sRNA_5dpa24 | AAAUGCCUGCAAAGAAGAAGACAG | 24 | 6 | At5g64520.1 (1); At5g64520.2 (1); At1g05200.1 (2); At2g22230.1 (2.5 y); At3g19830.1 9 (2.5); At1g31300.1 (3); At2g12905.1 (3); At3g03790.1 (3); At3g03790.2 (3); At5g24380.1 (3); At3g60930.1 (3); At4g06603.1 (3); At1g73630.1 (3); At4g05220.1 (3) | glutamate receptor family protein (GLR3.4) plant glutamate receptor family; expressed protein(3); calcium-binding protein; beta-hydroxyacyl-ACP dehydratase; ankyrin repeat family protein / regulator of chromosome condensation (RCC1) family protein (similar to hect domain and RLD 2(2); C2 domain-containing protein; harpin-induced protein-related / HIN1-related / harpin-responsive protein-related; transporter (putative similar to iron-phytosiderophore transporter protein yellow stripe 1); DNA repair protein-related (DNA-repair protein XRCC2-like)(2) |
| Gh_sRNA_5dpa16 | AUCUAGUUGGACUGCCACGUAGGA | 24 | 5 | At2g35270.1 (2); At4g04040.1 (2.5); At3g62170.1 (3) | pyrophosphate--fructose-6-phosphate 1-phosphotransferase beta subunit; DNA-binding protein-related; pectinesterase family protein; |
| Gh_sRNA_5dpa45 | CCCAAGAAUAACAUCUGCUACA | 22 | 5 | At4g32180.1 (2); At3g13330.1 (3); At3g61750.1 (3); At5g25130.1 (3); At5g47140.1 (3); At1g60890.1 (3) | cytochrome P450 family protein CYTOCHROME P450 71B1; auxin-responsive protein-related (similar to auxin-induced protein AIR12); eukaryotic pantothenate kinase family protein; zinc finger (GATA type) family protein; phosphatidylinositol-4-phosphate 5-kinase family protein; |
| Gh_sRNA_5dpa46 | AAAGACUUGGUAUUUAAGAGAU | 22 | 5 | At2g36350.1 (2.5); At3g48060.1 (2.5); At1g05010.1 (3); At1g32300.1 (3); At1g69940.1 (3); At3g04210.1 (3); At3g56610.1 (3);At5g21900.1 (3); At5g27030.1 (3); At2g34660.1 (3); At3g46530.1 (3); At3g48050.1 (3); At3g48050.2 (3) | protein kinase (putative similar to protein kinase KIPK (KCBP-interacting protein kinase); disease resistance protein, RPP13-like (CC-NBS class); FAD-binding domain-containing protein; disease resistance protein (TIR-NBS class); expressed protein(2); pectinesterase family protein; WD-40 repeat family protein; glutathione S-conjugate ABC transporter (MRP2) (almost identical to MgATP-energized glutathione S-conjugate pump); bromo-adjacent homology (BAH) domain-containing protein(3); 1-aminocyclopropane-1-carboxylate oxidase / ACC oxidase / ethylene-forming enzyme (ACO) (EAT1) |
| **6 dpa** |  |  |  |  |  |
| Gh_sRNA_6dpa11 | GUUACGCACGAGGAAGAGUUAGCA | 24 | 50 | At1g34640.1 (2.5); At1g27180.1 (3) | disease resistance protein (TIR-NBS-LRR class); expressed protein |
| Gh_sRNA_6dpa03 | AUAUAUCUCCAAUCAUGACCGCCC | 24 | 37 | At2g18040.1(2.5); At1g04220.1 (3); At2g12700.1 (3); At1g13900.1 (3); At1g48840.1 (3); At2g29980.2 (3); At4g12240.1 (3); At5g48020.1 (3); At5g61740.1 (3); At1g43800.1 (3) | omega-3 fatty acid desaturase, endoplasmic reticulum (FAD3)(2); peptidyl-prolyl cis-trans isomerase (PIN1) / cyclophilin / rotamase; ABC transporter family protein; beta-ketoacyl-CoA synthase; expressed protein; zinc finger (C2H2 type) family protein(2); acyl-[acyl-carrier-protein] desaturase (putative stearoyl-ACP desaturase); calcineurin-like phosphoesterase family protein |
| Gh_sRNA_6dpa16 | CAUGCCCAUGAGAUUAUUUAUCA | 23 | 19 | At1g77310.1 (2.5); At4g25660.1 (2.5); At3g52230.1 (3); At4g12100.1 (3); At1g29020.1 (3); At3g47110.1 (3) | calcium-binding EF hand family protein; wound-responsive protein; leucine-rich repeat transmembrane protein kinase; expressed protein(2); expressed protein cullin-4A |
| Gh_sRNA_6dpa05 | AAAACUAAACCCCGAAAACCCGAA | 24 | 17 | At1g75980.1(3); At4g15415.2 (3); At1g77480.2 (3); At2g39190.2 (3); At3g17840.1(3) | ABC1 family protein(2); nucellin protein (putative similar to nucellin (similar to aspartic protease)(2); serine/threonine protein phosphatase 2A (PP2A) regulatory subunit B' (B'gamma)(2); expressed protein |
| Gh_sRNA_6dpa09 | UUUUUCACUGUCCAAGGUAAGCCU | 24 | 17 | At1g43245.1 (2); At5g14510.1 (2.5); At1g65150.1 (2.5); At4g13430.1 (2.5); At2g03140.1 (2.5); At5g50360.1 (2.5); At1g65050.1 (3); At2g25050.1 (3); At09790.1 (3); At4g23440.1 (3); At23180.1 (3); At2g35330.1 (3); At3g07960.1 (3); At4g19510.1 (3); At4g14980.1 (3); At1g27710.1 (3); At4g38600.1 (3) | aconitase family protein / aconitate hydratase family protein; armadillo/beta-catenin repeat family protein(2); CAAX amino terminal protease family protein; DC1 domain-containing protein; disease resistance protein (TIR-NBS-LRR class); expressed protein(3); formin homology 2 domain-containing protein / FH2 domain-containing protein; glycine-rich protein; HECT-domain-containing protein / ubiquitin-transferase family protein(2); meprin and TRAF homology domain-containing protein / MATH domain-containing protein (similar to ubiquitin-specific protease 12)(3); phosphatidylinositol-4-phosphate 5-kinase family protein; polyubiquitin (UBQ8); zinc finger (C3HC4-type RING finger) protein-related |
| Gh_sRNA_6dpa26 | AAGAGAAGAGAAUGUGAGC | 19 | 16 | none | none |
| Gh_sRNA_6dpa07 | AGAAGCUAGAGACAUAAAUGUGAC | 24 | 13 | At4g16340.1 (3); At1g66190.1 (3); At3g28750.1 (3); At5g04560.1 (3); At1g71220.1 (3); At2g32275.1 (3); At2g20790.3 (3); At5g65590.1 (3) | UDP-glucose:glycoprotein glucosyltransferase; Dof-type zinc finger domain-containing protein; expressed protein expression supported by MPSS; expressed protein; DEMETER protein (DME); adapter protein SPIKE1 (SPK1); expressed protein |
| Gh_sRNA_6dpa08 | UUUGGAGUACAUUCCACCUGCUGU | 24 | 12 | At1g66950.1 (3); At2g36380.1 (3); At4g01770.1 (3); At4g21430.1 (3); | ABC transporter family protein; hypothetical protein; ABC transporter family protein related to multi drug resistance proteins and P-glycoproteins; transcription factor jumonji (jmjC) domain-containing protein |
| Gh_sRNA_6dpa04 | AUCGAUUGACAUAGACAUUAUUGU | 24 | 11 | At5g48485.1 (2.5); At3g05090.1 (3); At1g16840.2 (3); At1g27410.1 (3); At5g23940.1 (3); | expressed protein; transferase family protein (similar to anthranilate N-hydroxycinnamoyl/benzoyltransferase); protease inhibitor/seed storage/lipid transfer protein (LTP) family protein; DNA cross-link repair protein-related; transducin family protein / WD-40 repeat family protein (similar to uncharacterized KIAA1449 protein) |
| Gh_sRNA_6dpa13 | AGAGGUGUAAUGGAAUAGAGGUGU | 24 | 9 | At1g48510.1 (2.5); At4g12060.1 (2.5); At2g35780.1 (2.5); At2g40470.1 (3); At4g19040.1 (3); At1g52910.1 (3 ) | LOB domain protein 15 / lateral organ boundaries domain protein 15 (LBD15) (similar to ASYMMETRIC LEAVES2); cytochrome c oxidase assembly protein; expressed protein; serine carboxypeptidase S10 family protein (similar to Serine carboxypeptidase II chains A and B); pleckstrin homology (PH) domain-containing protein / lipid-binding START domain-containing protein; Clp amino terminal domain-containing protein |
| Gh_sRNA_6dpa25 | CAUCCGGGGGAAGAAUGGCA | 20 | 9 | none | none |
| Gh_sRNA_6dpa06 | GAAGCCAGAGCGAUUCUUGAAGGA | 24 | 5 | At2g26350.1 (2.5); At3g07020.1 (2.5); At5g18780.1 (2.5); At5g06860.1 (3); At3g17890.1 (3); At2g45350.1 (3); At5g59390.1 (3) | pentatricopeptide (PPR) repeat-containing protein; zinc-binding peroxisomal integral membrane protein (PEX10); F-box family protein; XH/XS domain-containing protein; UDP-glucose:sterol glucosyltransferase (UGT80A2); expressed protein; polygalacturonase inhibiting protein 1 (PGIP1) |
| **7 dpa** |  |  |  |  |  |
| Gh_sRNA_7dpa05 | AGAACUCACCAAGCCUAAGGAUUG | 24 | 144 | At5g55540.1 (3); At2g19340.1 (3); At5g40240.1 (3) | membrane protein; nodulin MtN21 family protein; expressed protein |
| Gh_sRNA_7dpa11 | AGAAAGCUCAAGGAGAAAGGAAAG | 24 | 16 | At2g23760.1 (2); At5g66390.1 (2.5); At2g36430.1 (2.5); At30515.1 (2.5); At2g22540.1 (2.5); At3g59040.2 (2.5); At4g01430.1 (2.5); At1g09870.1 (2.5); At3g56880.1 (3); At5g08310.1 (3); At1g33230.1 (3); At1g66780.1 (3); At1g68940.1 (3); At3g54350.2 (3); At4g39450.1 (3); At5g11970.1 (3); At5g53250.1 (3); At1g11840.1 (3); At2g17980.1 (3); At4g20800.1 (3); At4g25680.1 (3); At5g11300.1 (3); At1g47580.1 (3); At4g24150.1 (3); At5g59510.1 (3) | arabinogalactan-protein; armadillo/beta-catenin repeat protein-related / U-box domain-containing protein; BEL1-like homeobox 4 protein (BLH4)(2); cyclin (similar to cyclin 3a, (CYC3b)); expressed protein(7); expression supported by MPSS; FAD-binding domain-containing protein (similar to Reticuline oxidase precursor, peroxidase 72 (PER72) (P72) (PRXR8)); forkhead-associated domain-containing protein / FHA domain-containing protein MSP58 - nucleolar protein; histidine acid phosphatase family protein (similar to multiple inositol polyphosphate phosphatase); lactoylglutathione lyase (putative glyoxalase I)(2); lipoyltransferase; MATE efflux family protein; nodulin MtN21 family protein; pentatricopeptide (PPR) repeat-containing protein (2); sec1 family protein; short vegetative phase protein (SVP); VQ motif-containing protein; |
| Gh_sRNA_7dpa06 | AUCAAGGGGGUGCCAGAUACGACA | 24 | 11 | At1g67120.1 (2.5); At4g09970.1 (3); At1g04970.1 (3) | lipid-binding serum glycoprotein family protein(2); midasin-related similar to Midasin (MIDAS-containing protein); expressed protein |
| Gh_sRNA_7dpa13 | ACACACCUGUUUGMGACGAUAUGC | 24 | 6 | At3g42910.1 (2.5); At5g59190.1 (2.5) | expressed protein; expression supported by MPSS; subtilase family protein |
| **8 dpa** |  |  |  |  |  |
| Gh_sRNA_8dpa10 | AUUGAGAAUCUAAACUUGGCCGCA | 24 | 47 | At1g55050.1 (2); At5g04895.1 (2.5); At4g23810.1 (2.5); At1g08150.1 (3); At1g60680.1 (3); At5g54180.1 (3); At5g60710.1 (3); At1g24350.1 (3); At5g64120.1 (3); At1g66140.1 (3); At2g38670.1 (3); At2g43040.1 (3); At3g22790.1 (3) | zinc finger (C2H2 type) family protein; kinase interacting family protein (similar to kinase interacting protein 1); zinc finger (C3HC4-type RING finger) family protein; WRKY family transcription factor AR411; helicase domain-containing protein (similar to DEIH-box RNA/DNA helicase); expressed protein ; expression supported by MPSS; expressed protein; peroxidase; ethanolamine-phosphate cytidylyltransferase; mitochondrial transcription termination factor-related / mTERF-related; sodium/hydrogen exchanger family protein; calmodulin-binding protein (similar to pollen-specific calmodulin-binding protein MPCBP) |
| Gh_sRNA_8dpa12 | GACUAAAAUGAAACCUAGGUGACU | 24 | 20 | At1g03170.1 (3); At1g47320.1 (3); At1g48090.1 (3); At1g48090.2 (3) | C2 domain-containing protein(2); expressed protein(2); |
| Gh_sRNA_8dpa11 | CAGCCCUUUGUCGCUUCGAUUCGU | 24 | 14 | At2g46250.1 (3); At5g49850.1 (3) | myosin heavy chain-related; jacalin lectin family protein (similar to myrosinase-binding protein homolog) |
| Gh_sRNA_8dpa13 | ACUUCCGCUAGCUCGUUGAAACUA | 24 | 14 | At5g01950.1 (2.5 y); At2g26840.1 (2.5); At4g25835.1 (2.5); At2g21380.1 (2.5); At2g45110.1 (2.5); At3g13490.1 (2.5); At2g42480.1 (2.5); At1g30450.1 (3); At1g30450.2 (3); At1g30450.3 (3); At3g07290.1 (3); At4g20890.1 (3) | Cation-chloride cotransporter; kinesin motor protein-related(3); AAA-type ATPase family protein; leucine-rich repeat transmembrane protein kinase; beta-expansin; expressed protein; meprin and TRAF homology domain-containing protein / MATH domain-containing protein (similar to ubiquitin-specific protease 12); tubulin beta-9 chain (TUB9); pentatricopeptide (PPR) repeat-containing protein; tRNA synthetase class II (D, K and N) family protein (similar to Lysyl-tRNA synthetase) |
| Gh_sRNA_8dpa06 | AGGUCAUGAGAGGCCCACAUGAGC | 24 | 11 | At2g31740.1 (2); At5g53510.1 (3) | oligopeptide transporter OPT family protein (similar to SP|P40900 Sexual differentiation process protein isp4); expressed protein |
| Gh_sRNA_8dpa27 | GCACGUCUGCCUGGGUGUCACGC | 23 | 9 | none | none |
| Gh_sRNA_8dpa07 | AGAUUCAUGCAAACACCCGAAAAU | 24 | 7 | At4g11290.1 (3); At3g13770.1 (3) | peroxidase; pentatricopeptide (PPR) repeat-containing protein |
| Gh_sRNA_8dpa16 | AAGACAACCGCGAGAGGAGAUUGC | 24 | 7 | At1g12500.1 (3); At3g07960.1 (3); At4g28670.1 (3 y); At4g23690.1 (3) | phosphate translocator-related; protein kinase family protein; phosphatidylinositol-4-phosphate 5-kinase family protein; disease resistance-responsive family protein / dirigent family protein (similar to disease resistance response protein 206-d) |
| Gh_sRNA_8dpa05 | AUCACCCAGCACCCGGGACUCACU | 24 | 6 | At3g51550.1 (2) | protein kinase family protein |
| Gh_sRNA_8dpa09 | AUGUCCUGAGGAAUGCAAAGAAGC | 24 | 6 | At1g22150.1 (1.5 y); At1g78000.1 (1.5 y); At1g78000.2 (1.5 y); At4g08620.1 (2 y); At1g77990.1 (2.5 y); At3g21250.1 (2.5 y); At2g20080.1 (3); At3g01310.1 (3); At3g59080.1 (3); At5g36240.1 (3) | zinc knuckle (CCHC-type) family protein; sulfate transporter; aspartyl protease family protein (similary to CND41, chloroplast nucleoid DNA binding protein); ABC transporter family protein (similar to MRP-like ABC transporter); sulfate transporter; expressed protein; sulfate transporter (Sultr1 &2)(2); sulfate transporter (Sultr1&3); expressed protein |
| Gh_sRNA_8dpa26 | UAAUUACCUUUUUCACCAUUCCA | 23 | 6 | At3g12900.1 (1.5); At2g41520.1 (2); At2g41520.2 (2); At3g14810.1 (2); At2g44880.1 (2.5 y); At1g05950.1 (2.5); At2g15660.1 (2.5); At4g20300.1 (2.5); At5g12870.1 (3); At5g56510.1 (3); At5g64640.1 (3) ; At1g02630.1 (3); At2g02080.1 (3 y); At4g13740.1 (3); At2g46560.1 (3 y); At1g24190.1 (3); At3g27010.1 (3) | DNAJ heat shock N-terminal domain-containing protein(2); equilibrative nucleoside transporter; expressed protein(3); hypothetical protein; mechanosensitive ion channel domain-containing protein / MS ion channel domain-containing protein; myb family transcription factor (MYB46); oxidoreductase, 2OG-Fe(II) oxygenase family protein (similar to 1-aminocyclopropane-1-carboxylate oxidase homolog (Protein E8)), desacetoxyvindoline-4-hydroxylase); paired amphipathic helix repeat-containing protein (similar to transcription co-repressor Sin3) pectinesterase family protein; pentatricopeptide (PPR) repeat-containing protein; pumilio/Puf RNA-binding domain-containing protein (contains similarity to RNA-binding protein); TCP family transcription factor; transducin family protein / WD-40 repeat family protein; zinc finger (C2H2 type) family protein |
| Gh_sRNA_8dpa28 | CUACAUGCUCAAAGAUUCAAGGU | 23 | 6 | At2g22010.1 (1.5 y); At3g47836.1 (2.5); At5g41700.3 (2.5); At2g05920.1 (2.5); At5g38350.1 (3 y); At1g04240.1 (3); At4g40030.1 (3); At5g16500.1 (3) | ubiquitin-conjugating enzyme 8 (UBC8) E2; subtilase family protein (similarity to cucumisin-like serine protease); zinc finger (C3HC4-type RING finger) family protein; auxin-responsive protein / indoleacetic acid-induced protein 3 (IAA3); expressed protein; protein kinase family protein; histone H3.2; disease resistance protein (NBS-LRR class) |
| Gh_sRNA_8dpa30 | UUUAGCCUCGAAAUUUACAAGU | 22 | 6 | At4g13430.1 (2.5); At5g19830.1 (3); At1g66210.1 (3); At2g34820.1 (3); At2g41790.1 (3); At1g05790.1 (3) | lipase class 3 family protein; subtilase family protein; peptidase M16 family protein / insulinase family protein; basic helix-loop-helix (bHLH) family protein; aconitase family protein / aconitate hydratase family protein; peptidyl-tRNA hydrolase family protein |
| Gh_sRNA_8dpa02 | AUUUUGCUCAAGCCUGGCCUACAAA | 25 | 5 | At3g13980.1 (2.5); At1g21050.1 (3); At3g49790.1 (3); At4g29790.1 (3) | expressed protein (4) |
| Gh_sRNA_8dpa29 | CCGCCUAAAAUUUGAGAAGGUGU | 23 | 5 | At4g17420.1 (2); At4g38620.1 (2.5); At5g58970.1 (3); At5g58970.2 (3); At2g36340.1 (3); At5g09670.1 (3) | loricrin-related; myb family transcription factor (MYB4); expressed protein; uncoupling protein (UCP2)(2); DNA-binding storekeeper protein-related |
| **9 dpa** |  |  |  |  |  |
| Gh_sRNA_9dpa01 | CAGCAAUGAGCCAACUUUGGUUCGCGAUUCGUUCGUUCC | 39 | 29 | At1g59830.1 (2.5); At1g59830.2 (2.5); At4g39080.1 (2.5); At2g37650.1 (3); At3g48230.1 (3); At1g19490.1 (3); At3g05970.1 (3); At3g53780.1 (3); At3g53780.2 (3) | Scarecrow-like transcription factor 9 (SCL9); expressed protein; serine/threonine protein phosphatase PP2A-2 catalytic subunit (PP2A2); long-chain-fatty-acid--CoA ligase / long-chain acyl-CoA synthetase (LACS6)(2); vacuolar proton ATPase; rhomboid family protein(2); bZIP transcription factor family protein |
| Gh_sRNA_9dpa03 | CUAGAGAAGAUAAUGAUGAUGCU | 23 | 11 | At3g62660.1 (1); At3g04850.1 (2); At4g37160.1 (2); At1g51170.1 (2.5); At1g64810.1 (2.5); At3g29060.1 (2.5); At1g31280.1 (2.5); At1g34370.1 (2.5); At1g34370.2 (2.5); At3g04560.1 (2.5); At3g43750.1 (2.5); At4g31040.1 (2.5); At1g66090.1 (2.5); At1g68390.1 (2.5); At2g44760.1 (2.5); At5g56190.2 (2.5); At1g70780.1 (2.5); At4g16920.1 (3); At4g16950.1 (3); At4g16950.2 (3); At1g16900.1 (3); At4g16890.1 (3); At4g32600.1 (3); At1g80440.1 (3 y); At2g19660.1 (3); At3g04000.1 (3); At5g46400.1 (3); At5g67410.1 (3); At1g04240.1 (3); At1g26510.1 (3 y); At1g54710.1 (3); At1g64450.1 (3); At2g01690.1 (3); At2g01690.2 (3); At2g16230.1 (3); At2g17520.1 (3); At2g30990.1 (3); At2g38830.1 (3); At3g26810.1 (3); At3g57480.1 (3); At4g20160.1 (3); At4g40050.1 (3); At5g66450.1 (3); At2g45290.1 (3); At3g45030.1 (3); At5g38740.1 (3); At5g44190.1 (3); At2g30700.1 (3) | Expressed protein contains 3 WD-40 repeats; disease resistance protein (TIR-NBS-LRR class)(4); expressed protein (9); hypothetical protein; multi-copper oxidase type I family protein; zinc finger (C2H2 type) family protein(2); protein kinase family protein / Ire1 homolog-2 (IRE1-2); tesmin/TSO1-like CXC domain-containing protein; EXS family protein / ERD1/XPR1/SYG1 family protein; proline-rich family protein; phosphatidic acid phosphatase-related / PAP2-related; zinc finger (C3HC4-type RING finger) family protein(2); short-chain dehydrogenase/reductase (SDR) family protein; zinc finger (C2H2 type, AN1-like) family protein; tumor susceptibility protein-related; auxin-responsive protein / indoleacetic acid-induced protein 3 (IAA3); kelch repeat-containing F-box family protein; myb family transcription factor (GLK2); DC1 domain-containing protein; curculin-like (mannose-binding) lectin family protein; repeat family protein contains 3 (2 significant) WD-40 repeats (similar to beta transducin-like protein HET-E2C*40); expressed protein (expression supported by MPSS)(2); F-box family protein contains F-box domain; transketolase (transketolase 1); MADS-box family protein; 40S ribosomal protein S20 (RPS20A) 40S ribsomomal proteinS20; protein kinase family protein; PAZ domain-containing protein / piwi domain-containing protein (similar to Argonaute protein (AGO1); glycosyl transferase family 8 protein; glycosyl hydrolase family 17 protein (similar to elicitor inducible chitinase Nt-SubE76); proton extrusion protein-related; transport inhibitor response protein (putative E3 ubiquitin ligase SCF complex F-box subunit); zinc finger (C2H2 type) family protein |
| **10 dpa** |  |  |  |  |  |
| Gh_sRNA_10dpa11 | GAGACCCCAUUGUAUGCCGGUGUC | 24 | 31 | TC34418 (0); CD486717 (1.5); TC38438 (2.5) | Annotation not available; weakly similar to Caffeic acid O-methyltransferase(2); similar to 30S ribosomal protein S5 |
| Gh_sRNA_10dpa02 | UUUUUCACUGUCCAAGGUAAGCCU | 24 | 20 | At1g23180.1 (3 y); At2g03140.1 (2.5); At4g38600.2 (3 y); At1g65150.1 (2.5); At4g14980.1 (3 y); At1g65050.1 (3); At1g27710.1 (3); At2g35330.1 (3); At1g43245.1 (2); At4g13430.1 (2.5); At5g14510.1 (2.5); At2g25050.1 (3 y); At3g07960.1 (3); At4g23440.1 (3 y); At4g38600.1 (3 y); At3g09790.1 (3); At1g65150.2 (2.5); At4g19510.1 (3 y); At5g50360.1(2.5) | HECT-domain-containing protein / ubiquitin-transferase family protein (similar to Thyroid receptor interacting protein 12 (TRIP12))(2); meprin and TRAF homology domain-containing protein / MATH domain-containing protein (similar to ubiquitin-specific protease 12)(3); DC1 domain-containing protein; glycine-rich protein; zinc finger (C3HC4-type RING finger) protein-related; expressed protein(3); aconitase family protein / aconitate hydratase family protein; armadillo/beta-catenin repeat family protein; formin homology 2 domain-containing protein / FH2 domain-containing protein; phosphatidylinositol-4-phosphate 5-kinase family protein; polyubiquitin (UBQ8); disease resistance protein (TIR-NBS-LRR class) |
| Gh_sRNA_10dpa07 | AAGAGAAUAAUGAGCCUUCAGUGC | 24 | 14 | At2g40540.1 (2.5); At3g58830.1 (3); At1g27520.1 (3); At2g13810.1 (3) | glycoside hydrolase family 47 protein; potassium transporter (putative (KT2) identical to putative potassium transporter AtKT2p, strong similarity to potassium transporter HAK2p); haloacid dehalogenase (HAD) superfamily protein; aminotransferase class I and II family protein |
| Gh_sRNA_10dpa33 | UUCAACGCACUUGAACCCACAUC | 23 | 14 | TC40710 (2); TC37980 (2.5) | beta-tubulin; similar to GB|AAO42802.1; Annotation not available |
| Gh_sRNA_10dpa08 | ACGUUAUGCCCUGAUCUAGAGGUG | 24 | 13 | At1g63640.1 (3); At1g63640.2 (3) | kinesin motor protein-related C-terminal region (2); |
| Gh_sRNA_10dpa14 | UAUACCUUUAAAACGCGAUCGAUU | 24 | 9 | TC33867 (2.5); BG443850 (3); TC29945 (3) | similar to GB|AAO64806.1; similar to aspartate aminotransferase; similar to Aspartate aminotransferase 2 |
| Gh_sRNA_10dpa34 | UAAUUUGAAAAGUGACUUUGAAC | 23 | 9 | At4g22700.1 (2.5); At1g63300.1 (3); At5g39210.1 (3); At1g21910.1 (3); At1g48260.1 (3); At1g52370.1 (3); At1g08760.1 (3); At1g27900.1 (3); At1g52830.1 (3); At3g51990.1 (3) | RNA helicase (putative similar to ATP-dependent helicase DDX8 (RNA helicase HRH1) (DEAH-box protein 8)); AP2 domain-containing transcription factor family protein (similar to TINY); expressed protein (similar to Intracellular protein transport protein USO1); auxin-responsive protein / indoleacetic acid-induced protein 6 (IAA6); expressed protein(2); ribosomal protein L22 family protein; LOB domain family protein / lateral organ boundaries domain family protein (LBD32); CBL-interacting protein kinase 17 (CIPK17); protein kinase family proteinprotein |
| Gh_sRNA_10dpa15 | UUAAUAUGUAGACCUCCAUGGAGC | 24 | 8 | At2g04880.1 (2.5); At2g04880.2 (2.5); At2g22570.1 (3); At2g22570.2 (3); At4g29810.1 (3) | mitogen-activated protein kinase kinase (MAPKK) (MKK2) (identical to MAP kinase kinase 2); isochorismatase hydrolase family protein(2); WRKY family transcription factor (ZAP1)(2) |
| Gh_sRNA_10dpa13 | AUAGCCAGAUAAUGUGAAGUGCCA | 24 | 7 | At1g30810.1 (2); At2g27110.1 (3); At2g27110.2 (3); At5g46520.1 (3) | disease resistance protein (TIR-NBS-LRR class); far-red impaired responsive protein(2); transcription factor jumonji (jmj) family protein / zinc finger (C5HC2 type) family protein |
| Gh_sRNA_10dpa17 | GAACCUGUUUCUUUGGUGUUGUAG | 24 | 7 | At5g42950.1 (1); At2g35155.1 (2); At1g20960.1 (2); At5g01840.1 (2.5); At5g52980.1 (2.5); At2g16040.1 (2.5); At3g57400.1 (2.5); At1g35090.1 (3); At4g19300.1 (3); At5g36050.1 (3); At1g21580.1 (3); At1g50910.1 (3); At1g34410.1 (3); At2g36480.1 (3); At3g11900.1 (3); At3g50960.1 (3); At5g17790.1 (3); At5g61140.1 (3);  At1g50690.1 (3); At3g45010.1 (3) | amino acid transporter family; DEAD box RNA helicase (putative similar to ASC-1 complex subunit P200); expressed protein expression supported by MPSS; expressed protein(5); GYF domain-containing protein; hAT dimerisation domain-containing protein / transposase-related; hydroxyproline-rich glycoprotein family; hypothetical protein(2); ovate family protein; serine carboxypeptidase III; transcriptional factor B3 family protein / auxin-responsive factor AUX/IAA-related; U5 small nuclear ribonucleoprotein helicase; zinc finger (C2H2-type) family; zinc finger (Ran-binding) family protein; |
| Gh_sRNA_10dpa03 | AAACCCUUGACUGAGUUGGUGUCA | 24 | 6 | At3g18010.1 (2); At2g26460.1 (2.5); At5g38190.1 (2.5); At1g72500.1 (2.5); At3g09300.1 (3); At2g20050.1 (3); At3g27860.1 (3); At5g43810.1 (3); At5g62530.1 (3) | RED family protein (similar to Red protein (RER protein)); delta-1-pyrroline-5-carboxylate dehydrogenase (P5CDH); myosin heavy chain-related; PWWP domain-containing protein; protein phosphatase 2C; pinhead protein (PINHEAD) / zwille protein (ZWILLE); oxysterol-binding family protein; homeobox-leucine zipper transcription factor family protein (similar to wuschel protein); inter-alpha-trypsin inhibitor heavy chain-related |
| Gh_sRNA_10dpa20 | AAAUCUUCGAACCUGAGUAGGUGA | 24 | 6 | At5g01370.1 (2.5); At2g27270.1 (2.5); At5g20410.1 (2.5); At3g16420.1 (3); At3g16420.2 (3); At3g16430.1 (3); At3g16430.2 (3); At3g58830.1 (3); At5g40250.1 (3); At1g31410.1 (3); At1g62020.1 (3); At4g05140.1 (3); At4g08450.1 (3 y); At4g33380.1 (3) | coatomer protein complex, subunit alpha; expressed protein(3); 1,2-diacylglycerol 3-beta-galactosyltransferase; (putative identical to monogalactosyldiacylglycerol synthase, similar to MGDG synthase type A); jacalin lectin family protein (similar to myrosinase binding protein)(4); putrescine-binding periplasmic protein-related (similar to Chain A, Putrescine Receptor (Potf)); haloacid dehalogenase (HAD) superfamily protein; disease resistance protein (TIR-NBS-LRR class); zinc finger (C3HC4-type RING finger) family protein (similar to RING-H2 finger protein RHX1a); equilibrative nucleoside transporter family protein (similar to nucleoside transporter FUN26) |
| Gh_sRNA_10dpa37 | UAUCCCGACCAUCCUAGCCGAG | 22 | 6 | At2g20610.1 (2); At2g20610.2 (2); At1g44990.1 (3); At2g37550.1 (3); At4g35580.1 (3) | no apical meristem (NAM) family protein (similar to TIP); aminotransferase (putative similar to nicotianamine aminotransferase)(2); hypothetical protein; arabidopsis pde1 suppressor 1 protein (ASP1) |
| Gh_sRNA_10dpa06 | UUUAUAUAAUUGGUGUGAAAGCGC | 24 | 5 | At1g79690.1 (3); At4g00150.1 (3); At2g44210.1 (3) | scarecrow-like transcription factor 6 (SCL6); expressed protein; MutT/nudix family protein |
| Gh_sRNA_10dpa12 | AGUGUCACGGAACAAAUGUCUUGA | 24 | 5 | At1g30475.1 (2.5); At1g62290.1 (3) | expressed protein; aspartyl protease family protein |
| Gh_sRNA_10dpa16 | GGAAAAGAGAUACAGAUGACGUGA | 24 | 5 | At1g51650.1 (2.5); At2g21340.1 (2.5); At2g21340.2 (2.5); At1g31760.1 (2.5); At3g24210.1 (2.5); At1g24190.1 (3); At1g69530.1 (3); At1g69530.2 (3); At1g69530.3 (3); At5g16900.1(3); At3g18040.2 (3) | ATP synthase epsilon chain, mitochondrial; mitogen-activated protein kinase (putative (MPK9)) (identical to ATMPK9); SWIB complex BAF60b domain-containing protein; enhanced disease susceptibility protein (putative / salicylic acid induction deficient protein, enhanced disease susceptibility 5)(2); ankyrin repeat family protein; expansin (putative (EXP1), identical to expansin (At-EXP1)); paired amphipathic helix repeat-containing protein (similar to transcription co-repressor Sin3); leucine-rich repeat protein kinase (similar to light repressible receptor protein kinase) |
| Gh_sRNA_10dpa25 | UAUACCAAUAAAACGCGAUCGAUU | 24 | 5 | At4g21300.1(3) | pentatricopeptide (PPR) repeat-containing protein |
| Gh_sRNA_10dpa26 | AAAGAUGACAUUGUUGGAAGGAAU | 24 | 5 | At1g19025.1 (2); At2g09910.1 (2); At2g27370.1 (2); At3g25290.1 (2.5); At1g26460.1 (2.5); At3g50130.1 (2.5); At5g27100.1 (2.5); At5g47470.1 (2.5); At1g79710.1 (2.5); At4g26850.1 (3); At5g55120.1 (3); At4g26340.1 (3); At5g15890.1 (3); At5g49750.1 (3 y); At1g75680.1 (3); At2g17420.1 (3); At3g28270.1 (3); At3g58510.1 (3); At3g58510.2 (3); At5g02030.1 (3); At1g13570.1 (3); At1g55720.1 (3); At1g55730.1 (3); At4g02070.1 (3); At4g37130.1 (3); At1g73560.1 (3); At3g26100.1 (3); At3g26100.2 (3); At3g60740.1 (3); At5g64320.1 (3) | auxin-responsive family protein (similar to auxin-induced protein AIR12); calcium exchanger (similar to low affinity calcium antiporter CAX2); cation exchanger, putative (CAX5) (similar to low affinity calcium antiporter CAX2, H+/Ca2+ exchanger 2); DEAD box RNA helicase (similar to RNA helicase DBY protein, DEAD-box protein 3(2); DNA cross-link repair protein-related; DNA mismatch repair protein MSH6-1; expressed protein (similar to At14a protein); expressed protein expression supported by MPSS; expressed protein(3); F-box family protein(2); glutamate receptor family protein (GLR2.1) (GLR3) plant glutamate receptor family; glycosyl hydrolase family 9 protein (similar to endo-beta-1,4-glucanase); homeodomain protein (BELLRINGER) several homeodomain proteins; hydroxyproline-rich glycoprotein family protein; hypothetical protein; integral membrane family protein (plant integral membrane protein); integral membrane transporter family protein (similar to high affinity folic acid/methotrexate transporter 5); leucine-rich repeat family protein; nodulin MtN21 family protein integral membrane protein; pentatricopeptide (PPR) repeat-containing protein(2); protease inhibitor/seed storage/lipid transfer protein (LTP) family protein; regulator of chromosome condensation (RCC1) family protein(2); thioredoxin reductase 2 / NADPH-dependent thioredoxin reductase 2 (NTR2); tubulin folding cofactor D; |
