# Supplementary material for: Small RNA regulation of ovule development in the cotton plant, G. hirsutum L
Source: BMC Plant Biol. 2008 Sep 16;8:93. doi: 10.1186/1471-2229-8-93 (PMC2564936; doi:10.1186/1471-2229-8-93)
Supplement: Additional file 6 — The schematic representation of fiber development stages and putatively targeted proteins by siRNAs of each 0–10 DPA ovules. [file 1471-2229-8-93-S6.pdf]

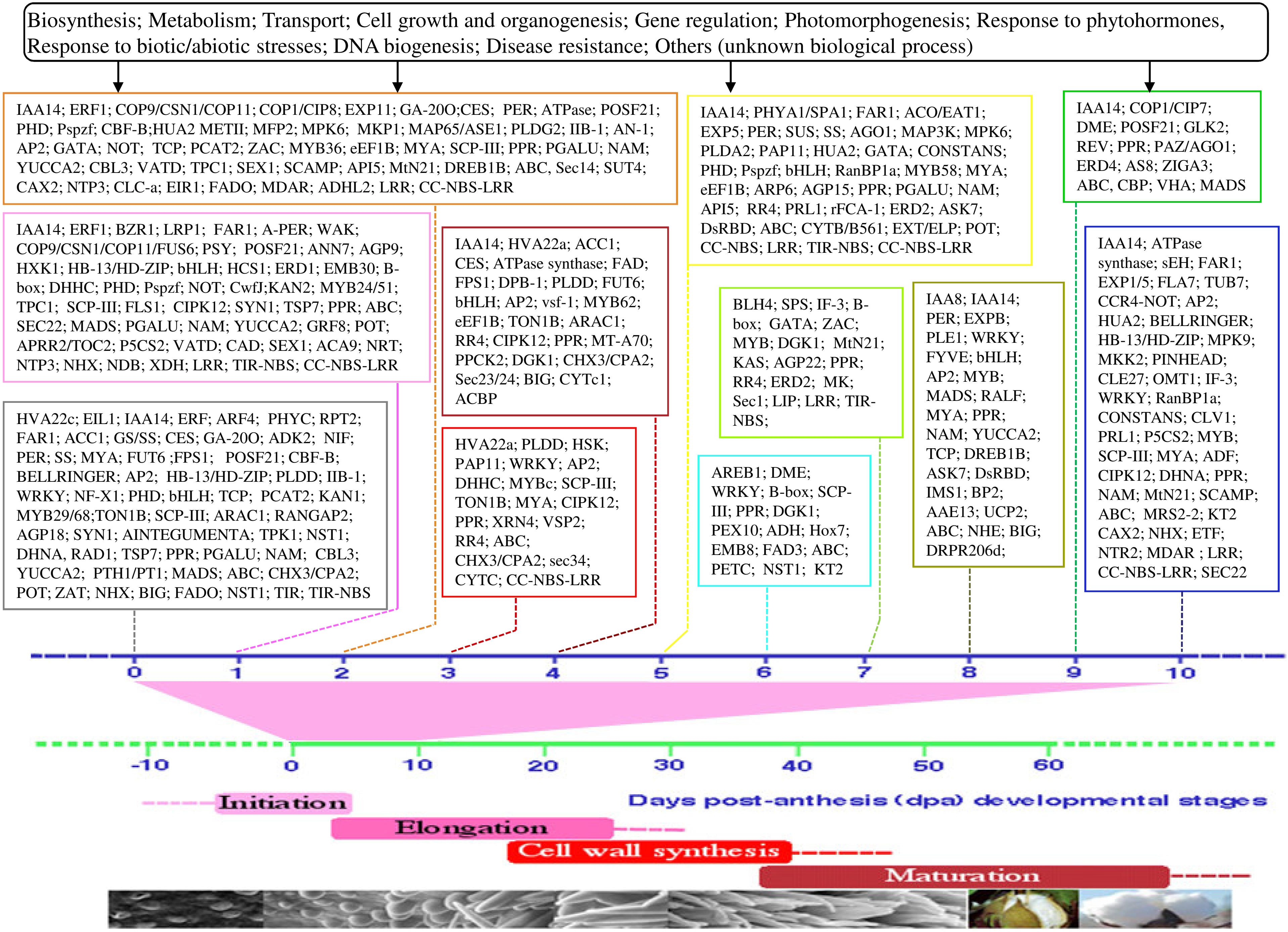

Biosynthesis; Metabolism; Transport; Cell growth and organogenesis; Gene regulation; Photomorphogenesis; Response to phytohormones, Response to biotic/abiotic stresses; DNA biogenesis; Disease resistance; Others (unknown biological process)
IAA14; ERF1; COP9/CSN1/COP11; COP1/CIP8; EXP11; GA-20O;CES; PER; ATPase; POSF21; PHD; Pspzf; CBF-B;HUA2 METII; MFP2; MPK6; MKP1; MAP65/ASE1; PLDG2; IIB-1; AN-1; AP2; GATA; NOT; TCP; PCAT2; ZAC; MYB36; eEF1B; MYA; SCP-III; PPR; PGALU; NAM; YUCCA2; CBL3; VATD; TPC1; SEX1; SCAMP; API5; MtN21; DREB1B; ABC, Sec14; SUT4; CAX2; NTP3; CLC-a; EIR1; FADO; MDAR; ADHL2; LRR; CC-NBS-LRR
IAA14; PHYA1/SPA1; FAR1; ACO/EAT1; EXP5; PER; SUS; SS; AGO1; MAP3K; MPK6; PLDA2; PAP11; HUA2; GATA; CONSTANS; PHD; Pspzf; bHLH; RanBP1a; MYB58; MYA; eEF1B; ARP6; AGP15; PPR; PGALU; NAM; API5; RR4; PRL1; rFCA-1; ERD2; ASK7; DsRBD; ABC; CYTB/B561; EXT/ELP; POT; CC-NBS; LRR; TIR-NBS; CC-NBS-LRR
IAA14; COP1/CIP7; DME; POSF21; GLK2; REV; PPR; PAZ/AGO1; ERD4; AS8; ZIGA3; ABC, CBP; VHA; MADS
IAA14; ERF1; BZR1; LRP1; FAR1; A-PER; WAK; COP9/CSN1/COP11/FUS6; PSY; POSF21; ANN7; AGP9; HXK1; HB-13/HD-ZIP; bHLH; HCS1; ERD1; EMB30; B-box; DHHC; PHD; Pspzf; NOT; CwfJ;KAN2; MYB24/51; TPC1; SCP-III; FLS1; CIPK12; SYN1; TSP7; PPR; ABC; SEC22; MADS; PGALU; NAM; YUCCA2; GRF8; POT; APRR2/TOC2; P5CS2; VATD; CAD; SEX1; ACA9; NRT; NTP3; NHX; NDB; XDH; LRR; TIR-NBS; CC-NBS-LRR
IAA14; HVA22a; ACC1; CES; ATPase synthase; FAD; FPS1; DPB-1; PLDD; FUT6; bHLH; AP2; vsf-1; MYB62; eEF1B; TON1B; ARAC1; RR4; CIPK12; PPR; MT-A70; PPCK2; DGK1; CHX3/CPA2; Sec23/24; BIG; CYTc1; ACBP
IAA14; ATPase synthase; sEH; FAR1; EXP1/5; FLA7; TUB7; CCR4-NOT; AP2; HUA2; BELLRINGER; HB-13/HD-ZIP; MPK9; MKK2; PINHEAD; CLE27; OMT1; IF-3; WRKY; RanBP1a; CONSTANS; CLV1; PRL1; P5CS2; MYB; SCP-III; MYA; ADF; CIPK12; DHNA; PPR; NAM; MtN21; SCAMP; ABC; MRS2-2; KT2 CAX2; NHX; ETF; NTR2; MDAR ; LRR; CC-NBS-LRR; SEC22
BLH4; SPS; IF-3; B-box; GATA; ZAC; MYB; DGK1; MtN21; KAS; AGP22; PPR; RR4; ERD2; MK; Sec1; LIP; LRR; TIR-NBS;
IAA8; IAA14; PER; EXPB; PLE1; WRKY; FYVE; bHLH; AP2; MYB; MADS; RALF; MYA; PPR; NAM; YUCCA2; TCP; DREB1B; ASK7; DsRBD; IMS1; BP2; AAE13; UCP2; ABC; NHE; BIG; DRPR206d;
HVA22c; EIL1; IAA14; ERF; ARF4; PHYC; RPT2; FAR1; ACC1; GS/SS; CES; GA-20O; ADK2; NIF; PER; SS; MYA; FUT6 ;FPS1; POSF21; CBF-B; BELLRINGER; AP2; HB-13/HD-ZIP; PLDD; IIB-1; WRKY; NF-X1; PHD; bHLH; TCP; PCAT2; KAN1; MYB29/68;TON1B; SCP-III; ARAC1; RANGAP2; AGP18; SYN1; AINTEGUMENTA; TPK1; NST1; DHNA, RAD1; TSP7; PPR; PGALU; NAM; CBL3; YUCCA2; PTH1/PT1; MADS; ABC; CHX3/CPA2; POT; ZAT; NHX; BIG; FADO; NST1; TIR; TIR-NBS
HVA22a; PLDD; HSK; PAP11; WRKY; AP2; DHHC; MYBc; SCP-III; TON1B; MYA; CIPK12; PPR; XRN4; VSP2; RR4; ABC; CHX3/CPA2; sec34; CYTC; CC-NBS-LRR
AREB1; DME; WRKY; B-box; SCP-III; PPR; DGK1; PEX10; ADH; Hox7; EMB8; FAD3; ABC; PETC; NST1; KT2
0 1 2 3 4 5 6 7 8 9 10
-10 0 10 20 30 40 50 60
Initiation
Days post-anthesis (dpa) developmental stages
Elongation
Cell wall synthesis
Maturation
