# Supplementary material for: Small RNA regulation of ovule development in the cotton plant, G. hirsutum L
Source: BMC Plant Biol. 2008 Sep 16;8:93. doi: 10.1186/1471-2229-8-93 (PMC2564936; doi:10.1186/1471-2229-8-93)
Supplement: Additional file 7 — The list of abbreviated putative target proteins (partial) used in Additional file 6 and the text. [file 1471-2229-8-93-S7.doc]

# Table S5. The list of abbreviated putative target proteins (partial) used in Figure S2 and the text

| **Abbreviation** | **Putative protein names** | **Abbreviation** | **Putative protein names** |
| --- | --- | --- | --- |
| AAE13 | Acyl-activating enzyme 13 | IIB-1 | Transcription initiation factor IIB-1 |
| ABA/AREB1 | ABA-responsive element-binding protein 1 | IMS1 | 2-isopropylmalate synthase 1 |
| ABC | ABC transporter | KAS | 3-ketoacyl-ACP synthase |
| ACA9 | Calcium-transporting ATPase, plasma membrane-type | KT2 | Potassium transporter |
| ACBP | Acyl-CoA binding family protein | LIP | Lipoyltransferase |
| ACC1 | Acetyl-CoA carboxylase 1 | LRP1 | Lateral root primordium 1 |
| ACO/EAT1 | 1-aminocyclopropane-1-carboxylate oxidase | MAP3K | MAP3K epsilon protein kinase |
| ADF | Actin-depolymerizing factor | MAP65/ASE1 | Microtubule associated protein family protein |
| ADH | Alcohol dehydrogenase | MDAR | Monodehydroascorbate reductase |
| ADK2 | Adenosine kinase 2 | METII | DNA (cytosine-5-)-methyltransferase |
| AGO1 | Argonaute protein | MFP2 | Fatty acid multifunctional protein |
| AGP | Arabinogallactan proteins | MK | Mevalonate kinase |
| AINTEGUMENTA | Ovule development protein | MKK2 | Mitogen-activated protein kinase kinase (MAPKK) |
| ALDH2 | Aldehyde dehydrogenase | MKP1 | MAP kinase phosphatase |
| AN-1 | Zinc finger (AN1-like) family protein | MPK | Mitogen activated protein kinase 6/9 |
| ANN7 | Annexin 7 | MRS2-2 | Magnesium transporter CorA-like family protein |
| AP2 | Floral homeotic protein APETALA2 | MT-A70 | Methyltransferase MT-A70 |
| A-PER | Anionic peroxidase | MtN21 | Nodulin MtN21 family protein |
| API5 | Apoptosis inhibitory 5 family protein | MYA | Myosin |
| APRR2/TOC2 | Pseudo-response regulator 2 | MYB | Myb family transcription factors (KAN1, KAN2, MYB24, MYB19, MYB36, MYB51, MYB58, MYB62, MYB68, MYBc, GLK2) |
| ARAC1 | Rac-like GTP-binding protein | NAM | No apical meristem family protein |
| ARF4 | Auxin responsive factor | NDB | Pyridine nucleotide-disulphide oxidoreductase family protein |
| ARP6 | Actin-related protein 6 | NF-X1 | Zinc finger (NF-X1 type) family protein |
| AS8 | Cadmium-responsive protein / cadmium induced protein | NHE | Sodium/hydrogen exchanger family protein |
| ASK7 | Shaggy-related protein kinase eta / ASK-eta 7 | NHX | Sodium proton exchanger |
| ASP1 | Arabidopsis pde1 suppressor 1 protein | NIF | NLI interacting factor family protein |
| B-box | Zinc finger (B-box type) family protein | NOT | Transcription regulator family protein (NOT2/NOT3/NOT5) |
| BELLRINGER | Homeodomain protein | NRT | High-affinity nitrate transporter |
| BGAL | Beta-galactosidase | NST1 | Nucleotide-sugar transport protein 1 |
| bHLH | Basic helix-loop-helix (bHLH) family protein | NTP3 | Nitrate transporter |
| BIG | Auxin transport protein | NTR2 | Thioredoxin reductase 2 |
| BLH4 | BEL1-like homeobox 4 protein | OMT1 | Quercetin 3-O-methyltransferase 1 |
| BP2 | Luminal binding protein 2 | P5CS2 | Delta 1-pyrroline-5-carboxylate synthetase B |
| BZR1 | Brassinosteroid signalling positive regulator-related | PAP11 | Purple acid phosphatase |
| CAD | Cinnamyl-alcohol dehydrogenase | PAZ/AGO1 | PAZ domain-containing protein |
| CAX5/CAX2 | Cation exchanger | PCAT2 | p300/CBP acetyltransferase-related protein 2 |
| CBF-B | CCAAT-binding transcription factor (CBF-B/NF-YA) family protein | PCT-BMYI | Beta-amylase PCT-BMYI |
| CBL3 | Calcineurin B-like protein 3 | PER | Peroxidase |
| CBP | Copper-binding family protein (similar to copper homeostasis) | PETC | Rieske [2Fe-2S] domain-containing protein |
| CCR4-NOT | CCR4-NOT transcription complex protein | PEX10 | Zinc-binding peroxisomal integral membrane protein |
| CES | Cellular development/orgonogenesisulose synthase family protein | PGAL | Polygalacturonase |
| CHX23/CPA2 | Cation/hydrogen exchanger | PHD | PHD finger family protein |
| CIPK12 | CBL-interacting protein kinase 12 | PHYA1/SPA1 | Phytochrome A supressor spa1 |
| CLC-a | Chloride channel protein | PHYC | Phytochrome C |
| CLE17 | putative CLAVATA3/ESR-Related 17 | PINHEAD | Pinhead protein / zwille protein (ZWILLE) |
| CLE27 | Putative CLAVATA3/ESR-Related 27 | PLDA2 | Phospholipase D alpha 2 |
| CLV1 | CLAVATA1 receptor kinase | PLDD | Phospholipase D delta |
| CMT1 | Chromomethylase 1 | PLDG2 | Phospholipase D gamma 2 |
| CONSTANS | Zinc finger CONSTANS-related | PLE1 | Pelota |
| COP1/CIP | COP1-interacting protein 7 and 8 | POSF21 | bZIP transcription factor |
| COP9/CSN1/COP11/FUS6 | COP9 signalosome complex subunit 1/CSN complex subunit 1/ COP11 protein/ FUSCA protein | POT | Proton-dependent oligopeptide transport family protein |
| CwfJ | CwfJ-like family protein | PPCK2 | Phosphoenolpyruvate carboxylase kinase 2 |
| CYTB/B561 | Cytochrome B/ Cytochrome B561 family | PPR | Pentatricopeptide |
| CYTC | Cytochrome C | PRL1 | PP1/PP2A phosphatases pleiotropic regulator 1 |
| CYTC1 | Cytochrome C1 | Pspzf | DNA binding zinc finger protein |
| DGK1 | Diacylglycerol kinase 1 | PSY | Phytoene synthase |
| DHHC | Zinc finger (DHHC type) family protein | PTH1/PT1 | Inorganic phosphate transporter |
| DHNA | Naphthoate synthase | RAD1/UVH1 | Repair endonuclease |
| DIN4 | branched-chain alpha-keto acid dehydrogenase E1 beta subunit | RALF | Rapid alkalinization factor family protein |
| DME | DNA glycosylase DEMETER protein | RanBP1a | Ran-binding protein 1a |
| DPB-1 | DPB-1 transcription factor | RANGAP2 | RAN GTPase activating protein 2 |
| DREB1B | DRE-binding protein | REV/IFL1 | Homeodomain-leucine zipper protein Revoluta/ fascicular fiberless 1 |
| DsRBD | Double-stranded RNA-binding domain containing protein | rFCA-1 | Flowering time control protein isoform |
| eEFA1B | Elongation factor 1B alpha-subunit 1 | RPT2 | Phototropic-responsive protein |
| EIL1 | Ethylene-insensitive 3-like1 | RR4 | Two-component responsive regulator |
| EIR1 | Auxin efflux carrier family protein | SAT-1 | Serine O-acetyltransferase |
| EMB30 | Pattern formation protein/GNOM-like | SCAMP | Secretory carrier membrane protein family protein |
| EMB8 | Embryogenesis-associated protein-related | SCP-III | Serine carboxypeptidase III |
| ERD1 | mitogen-activated protein kinase kinase kinase (MAPKKK) | Sec1 | sec1 family protein |
| ERD2 | ER lumen protein retaining receptor / HDEL receptor | Sec14 | SEC14 cytosolic factor |
| ERD4 | Early-responsive to dehydration protein-related | Sec23/24 | sec23/sec24 transport family protein |
| ERF1 | Ethylene-responsive factor 1 | sec34 | Secretory family protein |
| ETF | Electron transfer flavoprotein alpha subunit family protein | sEH | Epoxide hydrolase, soluble |
| EXL6 | GDSL-motif lipase | sel-12 | Presenilin family protein |
| EXP5/EXP11 | Expansin | SEX1 | Starch excess protein |
| EXPB | Beta-expansin | SGR2 | Shoot gravitropism 2 |
| EXT/ELP | Extensin/extensin like protein | SPS | Sucrose-phosphate synthase |
| FAD | Fatty acid desaturase family protein | SS | Starch synthase |
| FADO | FAD linked oxidase family protein | SUS | Sucrose synthase |
| FAR1 | Far-red impaired responsive family protein / FAR1 family protein | SUT4 | Sucrose transporter / sucrose-proton symporter |
| FLA7 | Fasciclin-like arabinogalactan-protein | SYN1 | Cohesion family protein SYN1 |
| FLS1 | Flavonol synthase 1 | TCP | TCP family transcription factor |
| FPS1 | Farnesyl pyrophosphate synthetase 1 | TON1B | Tonneau 1b |
| FUT6 | Xyloglucan fucosyltransferase | TPC1 | Two-pore calcium channel |
| FYVE | Zinc finger (FYVE type) family protein | TPK1 | Thiamin pyrophosphokinase |
| GA-20O | Gibberellin 20-oxidase | TPS7 | Trehalose-6-phosphate phosphatase |
| GATA | Zinc finger (GATA type) family protein | TUB7 | Beta-tubilin |
| GRF8 | 14-3-3 protein GF14 kappa | UCP2 | Uncoupling protein |
| GS/SS | Glycogen synthase | VATD | Vacuolar ATP synthase subunit D |
| GSH2 | Glutathione synthetase | VHA | Vacuolar proton ATPase |
| HB-13/HD-ZIP | Homeobox-leucine zipper protein 13 / HD-ZIP transcription factor 13 | vsf-1 | bZIP transcription factor |
| HCS1 | Holocarboxylase synthetase 1 | VSP2 | Vegetative storage protein 2 |
| Hox7 | Homeobox transcription factor | WAK | Wall associated kinase |
| HSK | Homoserine kinase | WRKY | WRKY family transcription factor |
| HUA2 | Putative PWWP domain-containing transcription factor | XDH | Xanthine dehydrogenase |
| HVA22 | ABA-responsive protein a and c | XRN4 | 5'-3' exoribonuclease |
| HXK1 | Hexokinase 1 | YUCCA2 | Flavin-containing monooxygenase |
| IAA14 | Auxin-responsive AUX/IAA family protein | ZAC | Zinc finger and C2 domain protein |
| IAA8 | Indoleacetic acid-induced protein 8 | ZAT | Zinc transporter |
| IF-3 | Translation initiation factor 3 | ZIGA3 | ARF GAP-like zinc finger-containing protein |
