# Supplementary material for: Small RNA regulation of ovule development in the cotton plant, G. hirsutum L
Source: BMC Plant Biol. 2008 Sep 16;8:93. doi: 10.1186/1471-2229-8-93 (PMC2564936; doi:10.1186/1471-2229-8-93)
Supplement: Additional file 8 — MirBase confirmed microRNAs functioning in different DPAs of cotton ovule development. * Blasted against GenBank (NCBI); TAIR (AGI and Higher plant EST databases); and Cotton Pilot Project (CPP) EST database; **In parentheses, the target scores and conservation (y) between A. thaliana and O. sativa genomes were given. [file 1471-2229-8-93-S8.doc]

# Table S6. MirBase confirmed microRNAs functioning in different DPAs of cotton ovule development

| **Small RNA name** | **MirBase match** | | **BLAST gene ID and score*** | **Name** | **Putative target protein ID** | **Target protein name** |
| --- | --- | --- | --- | --- | --- | --- |
| 0dpa103 | | miR172 | Gi|48738994 (e= 0.018. +/+); AT3G55512.1 (e = 7e-05 +/+) | G. raimondii cDNA; ath-miR172D | At4g36920.1 (0.5 y); At5g60120.1 (0.5 y); At2g28056.1 (1); At2g28550.1 (1.5 y); At2g28550.2 (1.5y); At5g67180.1 (1.5 y); At2g39250.1 (1.5 y); At2g10440.1 (2.5); At1g32340.1 (2.5 y) At1g21060.1 (3); At3g49690.1 (3); At5g12900.1 (3); At5g19560.1 (3); At5g61110.1 (3); At5g65790.1 (3); At2g17520.1 (3); At3g47360.1 (3); At5g09560.1 (3); At3g12590.1 (3); At3g14770.1 (3) | Floral homeotic protein APETALA2 (AP2); Hypothetical proteins; zinc finger (C3HC4-type RING finger); protein kinase family protein / Ire1 homolog-2 (IRE1-2); KH domain-containing protein; short-chain dehydrogenase/reductase (SDR); nodulin MtN3; expressed proteins; myb family transcription factor (MYB68) |
| 0dpa104 | | miR390 |  |  | At2g15420.1 (3); At5g03640.1 (3 y) | Myosin heavy chain protein; protein kinase family protein contains serine/threonine protein kinase domain |
| 1dpa84 | | miR390 | AT5G58465.1 (e= 7e-05, +/+); gi|109839591(e = 0.005, +/+); gi|109889195 (e= 0.005, +/+); gi|109889196 (e= 0.005, +/+) | MIR390B; G. hirsutum cDNAs | At5g03640.1 (2.5 y); At1g47890.1 (3 y); At5g49660.1 (3 y) | Disease resistance family protein contains leucine rich-repeat domains; protein kinase family protein contains serine/threonine protein kinase domain; leucine-rich repeat transmembrane protein kinase |
| 2dpa119 | | miR172 | AT4G36920.1 (e= 7e-05, +/-); gi|109845337 (e = 0.005, +/+); gi|11203047 (e=0.005, +/-); cpp|34285 (e = 3e-04, +/-); cpp|37274| (e= 0.001, +/+) | APETALA2 transcription factor; G. hirsutum cDNA; G. arboreum fiber library cDNAs | At4g36920.1 (0 y); At2g28550.1 (1 y); At2g28550.2 (1 y); At5g67180.1 (1 y); At2g39250.1 (1 y); At2g28056.1 (2.5); At3g12590.1 (2.5 ); At4g29430.1 (2.5); At4g37030.1 (3) | Floral homeotic protein APETALA2 (AP2); 40S ribosomal protein S15A (RPS15aE); expressed proteins; hypothetical proteins |
| 2dpa126 | | miR390 | AT5G58465.1 (e = 7e-05, +/+); gi|109889195 (e = 0.005, +/+); gi|109889195(e= 0.005; +/+); gi|109889196 (e= 0.005,+/-) | MIR390B; *G. hirsutum* cDNAs | At5g03640.1 (2.5 y); At1g47890.1 (3 y); At5g49660.1 (3 y) | Disease resistance family protein contains leucine rich-repeat domains; protein kinase family protein contains serine/threonine protein kinase domain; leucine-rich repeat transmembrane protein kinase |
| 3dpa18 | | ath-mir853-like (e=0.008) |  |  | At5g58370.1 (3); At5g58370.2 (3) | Unknown expressed proteins |
| 3dpa26 | | miR172 | cpp|34285 (e = 3e-04, +/-); cpp|37274 (e= 0.001, +/+); gi|11203047 (e = 0.005, +/-); AT4G36920.1(e = 7e-05, +/-) | *G. arboreum* fiber cDNAs; APETALA2 transcription factor | At4g36920.1 (0 y); At2g28550.1 (1 y); At2g28550.2 (1 y); At5g67180.1 (1 y); At2g39250.1 (1 y); At2g28056.1 (2.5) ; At3g12590.1 (2.5); At4g29430.1 (2.5); At4g37030.1 (3 ) | APETALA2 (AP2); 40S ribosomal protein S15A (RPS15aE); AP2 domain-containing transcription factor; expressed protein; hypothetical protein |
| 4dpa25 | | miR172 | AT5G60120.1 (e = 0.006, +/-) | APETALA2 transcription factor; TOE2 /TOE2 (TARGET OF EAT1/2) DNA binding transcription factor | At2g28550.1 ( 0.5 y); At2g28550.2 (0.5 y); At5g60120.1 (0.5 y); At4g36920.1 (1 y); At5g67180.1 (1 y); At1g72050.1 (2) ; At1g72050.2 (2) ; At5g03415.1 (2) ; At1g08660.1 (2.5); At1g08660.2 (2.5); At2g39250.1 (2.5 y); At5g37060.1 (2.5); At2g24285.1 (3); At3g21500.1 (3); At3g21500.2 (3); At5g03550.1 (3); At5g61710.1 (3); At2g28056.1 (3); At4g25430.1 (3); At5g10520.1 (3 y); At5g47770.1 (3); At1g14460.1 (3); At2g42650.1 (3); At3g19760.1 (3); At4g23950.1 (3); At5g53780.1 (3); At5g61020.1 (3); At5g61020.2 (3); At5g66790.1 (3 y); At3g04530.1 (3 y); At5g61160.1 (3); At3g04110.1 (3) | Phosphoenolpyruvate carboxylase kinase 2 (PPCK2); glycosyl transferase family 29 protein / sialyltransferase family protein; YT521-B-like family protein; 1-deoxy-D-xylulose 5-phosphate synthase, putative / 1-deoxyxylulose-5-phosphate synthase, putative / DXP-synthase; DPB-1 transcription factor zinc finger (C2H2 type); AP2 domain-containing transcription factor; transferase family protein; protein kinase family protein; hypothetical proteins; expressed protein; cation/hydrogen exchanger, putative (CHX24); glutamate receptor family protein (GLR1.1); 60S ribosomal protein; DNA polymerase III; eukaryotic translation initiation factor 4A, putative / eIF-4A, putative / DEAD box RNA helicase; farnesyl pyrophosphate synthetase 1 |
| 7dpa23 | | miR390 | AT5G58465.1 (e = 7e-05, +/+); gi|109889195 (e = 0.005, +/+); gi|109889195(e= 0.005; +/+); gi|109889196 (e= 0.005,+/-) | MIR390B; *G. hirsutum* cDNAs | At5g03640.1 (2.5 y); At1g47890.1 (3 y); At5g49660.1 (3 y) | Disease resistance family protein contains leucine rich-repeat domains; protein kinase family protein contains serine/threonine protein kinase domain; leucine-rich repeat transmembrane protein kinase |

* Blasted against GenBank (NCBI); TAIR (AGI and Higher plant EST databases); and Cotton Pilot Project (CPP) EST database; **In parentheses, the target scores and conservation (y) between *A. thaliana* and *O. sativa* genomes were given.
